# Pandemic preparedness improves national-level SARS-CoV-2 infection and mortality data completeness: a cross-country ecologic analysis

**DOI:** 10.1186/s12963-024-00333-1

**Published:** 2024-06-15

**Authors:** Jorge R. Ledesma, Irene Papanicolas, Michael A. Stoto, Stavroula A. Chrysanthopoulou, Christopher R. Isaac, Mark N. Lurie, Jennifer B. Nuzzo

**Affiliations:** 1 Department of Epidemiology, Brown University School of Public Health, 121 S Main St, Providence, RI 02912 USA; 2 Department of Health Services, Policy and Practice, Brown University School of Public Health, 121 S Main St, Providence, RI 02912 USA; 3 Department of Health Management and Policy, School of Health, Georgetown University, 3700 Reservoir Road, N.W., Washington, DC 20057 USA; 4 Department of Biostatistics, Brown University School of Public Health, 121 S Main St, Providence, RI 02912 USA; 5 Nuclear Threat Initiative, 1776 Eye Street, NW, Suite 600, Washington, DC 20006 USA; 6 International Health Institute, Brown University School of Public Health, 121 S Main St, Providence, RI 02912 USA; 7 Population Studies and Training Center, Brown University, 68 Waterman St., Box 1836, Providence, RI 02912 USA; 8 Pandemic Center, Brown University School of Public Health, 121 S Main St, Providence, RI 02912 USA

**Keywords:** COVID-19, SARS-CoV-2, Pandemic preparedness, Global health security, Data completeness

## Abstract

**Background:**

Heterogeneity in national SARS-CoV-2 infection surveillance capabilities may compromise global enumeration and tracking of COVID-19 cases and deaths and bias analyses of the pandemic’s tolls. Taking account of heterogeneity in data completeness may thus help clarify analyses of the relationship between COVID-19 outcomes and standard preparedness measures.

**Methods:**

We examined country-level associations of pandemic preparedness capacities inventories, from the Global Health Security (GHS) Index and Joint External Evaluation (JEE), on SARS-CoV-2 infection and COVID-19 death data completion rates adjusted for income. Analyses were stratified by 100, 100–300, 300–500, and 500–700 days after the first reported case in each country. We subsequently reevaluated the relationship of pandemic preparedness on SARS-CoV-2 infection and age-standardized COVID-19 death rates adjusted for cross-country differentials in data completeness during the pre-vaccine era.

**Results:**

Every 10% increase in the GHS Index was associated with a 14.9% (95% confidence interval 8.34–21.8%) increase in SARS-CoV-2 infection completion rate and a 10.6% (5.91–15.4%) increase in the death completion rate during the entire observation period. Disease prevention (infections: β = 1.08 [1.05–1.10], deaths: β = 1.05 [1.04–1.07]), detection (infections: β = 1.04 [1.01–1.06], deaths: β = 1.03 [1.01–1.05]), response (infections: β = 1.06 [1.00–1.13], deaths: β = 1.05 [1.00–1.10]), health system (infections: β = 1.06 [1.03–1.10], deaths: β = 1.05 [1.03–1.07]), and risk environment (infections: β = 1.27 [1.15–1.41], deaths: β = 1.15 [1.08–1.23]) were associated with both data completeness outcomes. Effect sizes of GHS Index on infection completion (Low income: β = 1.18 [1.04–1.34], Lower Middle income: β = 1.41 [1.16–1.71]) and death completion rates (Low income: β = 1.19 [1.09–1.31], Lower Middle income: β = 1.25 [1.10–1.43]) were largest in LMICs. After adjustment for cross-country differences in data completeness, each 10% increase in the GHS Index was associated with a 13.5% (4.80–21.4%) decrease in SARS-CoV-2 infection rate at 100 days and a 9.10 (1.07–16.5%) decrease at 300 days. For age-standardized COVID-19 death rates, each 10% increase in the GHS Index was with a 15.7% (5.19–25.0%) decrease at 100 days and a 10.3% (− 0.00–19.5%) decrease at 300 days.

**Conclusions:**

Results support the pre-pandemic hypothesis that countries with greater pandemic preparedness capacities have larger SARS-CoV-2 infection and mortality data completeness rates and lower COVID-19 disease burdens. More high-quality data of COVID-19 impact based on direct measurement are needed.

**Supplementary Information:**

The online version contains supplementary material available at 10.1186/s12963-024-00333-1.

## Background

The coronavirus disease 2019 (COVID-19) pandemic was an unprecedented global health emergency imposing extensive challenges on health systems, economies, and societies across the globe. However, the ability to track the impacts of COVID-19 at the global level is challenged by heterogenous country-specific approaches to surveillance. These differences have likely contributed to findings that global case detection is 7% [1] and global deaths are at least 3 times greater than reported deaths [2]. These suboptimal levels of detection have further contributed to the widespread and dynamic nature of the pandemic [3–6]: When large numbers of people with COVID-19 go undetected, they cannot be systematically isolated, silently expose a large fraction of the population to the disease, and derail costly pandemic responses. Undercounting of the true mortality impact further inhibits accurate assessments of national control strategy effectiveness [7].

Capacities for detection and enumeration are therefore essential components of pandemic preparedness and response [8]. One tool for assessing pandemic preparedness that has received a great deal of attention is the Global Health Security (GHS) Index–which measures countries’ capacity to carry out necessary functions for preventing, detecting, and responding to infectious disease threats–due to studies reporting a positive correlation between the index and reported COVID-19 outcomes [9]. These findings have been used to suggest that better prepared countries experience more cases and deaths than countries with lower measured levels of preparedness [10–14]. However, these studies have not been able to fully account for heterogeneity in the completeness and representative of COVID-19 case and death data across countries [15]. Countries that have stronger public health and surveillance systems, and subsequently score higher in pandemic preparedness indices, may have more capacities to track COVID-19 cases and deaths compared to countries with weaker systems. This phenomenon may have then induced potential differential outcome measurement error in prior assessments of pandemic preparedness with the development of unexpected correlations. Taking account of heterogeneity in data completeness thus may help clarify analyses of the relationship between COVID-19 outcomes and standard preparedness measures.

Analyses that adjust for cross-country differences in COVID-19 data completeness may subsequently observe a more accurate estimate of the contributions of pandemic preparedness in supporting COVID-19 responses. However, there remains other sources of biases that may limit the robustness of the findings. The primary limitation is owing to differences in population age structures across countries, which is a critical consideration for studies of COVID-19 death tolls as the latest WHO data suggests that 80% of all global COVID-19 deaths have occurred in people aged 60 years and older [16]. The substantially greater risk of COVID-19 mortality among older people may therefore cause countries with older populations to have greater COVID-19 deaths compared to those with younger populations. Rigorous reevaluations of pandemic preparedness on COVID-19 cases and deaths while considering country-level differences in COVID-19 data completeness and age-standardization methods may provide additional information on the contributions of pandemic preparedness in supporting effective COVID-19 responses.

Here we leverage available global data on total SARS-CoV-2 infections and mortality to investigate both the relationship between pandemic preparedness and COVID-19 data completeness, and reassess the preparedness–COVID-19 burden relationship after accounting for data completeness and age-structure. Leveraging available global data in combination with reported case and mortality statistics, we first computed rates of SARS-CoV-2 infection and mortality data completeness. We separately analyze infection and mortality data completeness because deaths may be easier to determine compared to infections as individuals may be asymptomatic or have limited access to healthcare. We then regress global health security and pandemic preparedness indices, and their individual components, with the data completeness metrics across different temporal periods in the pandemic. Finally, we reevaluate the preparedness–COVID-19 burden relationship after adjusting for country-level differentials in data completeness. These analyses may help inform pandemic preparedness and response policies by identifying mechanisms for improving disease surveillance to help prevent widespread transmission and mortality while illuminating potential biases in previous assessments of pandemic responses.

## Methods

### Data sources

We collected data on country-level preparedness against infectious threats from the 2021 Global Health Security (GHS) Index. The measurement quantifies country’s abilities or potential to carry out public health functions necessary for disease outbreak prevention, detection, and response. We further extracted data on the six individual categories that compromise the index (prevention, detection and reporting, rapid response, health system, compliance with international norms, risk environment). Since each GHS category contains various indicators and sub-indicators, we included a set of indicators identified a priori to help identify specific capacities that modulate the outcomes. The indices range from 0 to 100 with lower scores indicating weaker health system capacities and higher scores suggesting stronger health system capacities. Additional details of the GHS index are included in appendix pp. 3.

We extracted country-level data on total SARS-CoV-2 infections and deaths from the Institute for Health Metrics and Evaluation’s (IHME’s) modeled estimates. IHME has previously published their estimation strategies in detail [1, 2, 17]. We subsequently linked data on cumulative reported cases and deaths to derive metrics of COVID-19 case and death completeness from the John Hopkins University Data Repository [18].

As a secondary measure of pandemic preparedness, we collected data from the Joint External Evaluation (JEE) ReadyScore. The JEE, developed by the World Health Organization (WHO), evaluates participating countries’ ability to prevent, detect, and respond to emerging infectious disease threats using 19 domains. As another measure of data completeness that is not dependent on modeling, we extracted COVID-19 testing rates from the Our World in Data database [19].

### Outcome measurement

We linked reported cumulative COVID-19 cases with total infection data to compute SARS-CoV-2 infection completion rates by taking the quotient between the two measures. We recomputed the completion rates at the following time periods after the first reported case in each country to standardize for variability in epidemic timelines: 100 days, 100–300 days, 300–500 days, and 500–700 days after the first reported case. To examine whether improvements in infection data completion varied by preparedness levels, we computed ratios of completion rates at 500 to 700 days to 100 days. This may provide an indication of whether countries with greater capacities were able to improve their data completion rates throughout the pandemic.

We derived a similar measure of data completion for mortality by dividing cumulative reported COVID-19 deaths by total estimated COVID-19 deaths, defined as all deaths where individuals were actively infected with COVID-19 at the time of death, to obtain COVID-19 death data completion rates. Unlike for infections, we did not derive death completion rates at multiple time points because country-level completion rates did not vary across the pandemic in the data (Table S4). We therefore provide inferences for COVID-19 mortality completion rates using cumulative death data up to the end of 2021 (2020–2021 inclusive).

### Statistical analyses

We employed multivariable linear regressions to examine the associations of the GHS Index on both SARS-CoV-2 infection and death data completeness rates at the country-level. We stratified regressions by 100, 100–300, 300–500, and 500–700 days following the first reported COVID-19 case in each country for infection completeness analyses. Our two outcome measures were log-transformed to examine the relative impact of the predictors. We also log-transformed the GHS Index and corresponding indicators as there was a logarithmic relationship between the indicators and the outcome (Figure S2). Although the GHS Index is our primary exposure, we ran separate bivariate regressions for each indicator to examine the effects of each category independent on others. Pre-pandemic gross Domestic Product (GDP) per capita was included in each regression to account for potential confounding identified a priori. To adjust for GDP per capita as a confounder, we computed pre-pandemic country-specific averages of GDP per capita for each country from 2015 to 2019. Since by definition a confounder occurs prior to the exposure and outcome, we leveraged GDP in multiple pre-pandemic years as countries were building and tracking preparedness capacities in the years leading to the pandemic. The average GDP per capita rates were subsequently log-transformed prior to analyses. We further decomposed the effect of income by presenting stratified regression analyses by World Bank income groups. To adjust for potential heteroscedasticity, we constructed confidence intervals with robust standard errors. Effect sizes are reported as completion rate ratios with values greater than 1 indicating increases in data completeness and values less than 1 indicating decreases as a function of the exposure.

As a secondary analysis, we reevaluated the GHS-COVID burden relationship utilizing infection and mortality data adjusted for cross-country differences in data completeness. We conducted this analysis by using cumulative infection and mortality data from IHME, the denominators of our completion metrics, as inputs into multivariable linear regression analyses adjusted for income with GHS indicators as focal predictors. Due to the marked relationship between COVID-19 mortality and age [17, 20], we indirectly age-standardized COVID-19 mortality using a comparative mortality ratio (CMR) approach [21] (Appendix pp. 5). These secondary analyses were limited to 100 and 300 days after the first reported case and death in each country to capture the period before widespread vaccine distribution. To decompose how biases my impact the GHS-COVID burden relationship, we present analyses using reported COVID-19 statistics, data adjusted for differences in data completeness, and total COVID-19 death data that is age-standardized. These secondary analyses included log-average pre-pandemic GDP per capita in regression analyses.

### One-way sensitivity analyses

To examine the robustness of our results based on input data, we conducted several sensitivity analyses using other measures of data completion rates. We repeated all analyses using COVID-19 testing rates as another measure of infection completion that is not dependent on modeling. To examine the robustness of our time cutoffs, we repeated the infection completion analysis at 100 days, 100–300 days, 300–500 days, and 500–700 days after the first global COVID-19 case, and calendar days every subsequent 6 months starting in June 2020. For mortality completeness rates, we derived ratios of excess mortality to reported COVID-19 mortality, a metric previously to illustrate differences in capacities to diagnose COVID-19 mortality [2, 22–24]. This was repeated using excess mortality estimates from IHME [2], WHO [24], and The Economist [25] representing cumulative excess deaths up to the end of 2021. We also examined whether utilizing the 2019 iteration of the GHS Index changed results. Finally, we examined whether including the stringency index, as a measure of real-time COVID-19 mitigation measures which may affect COVID-19 burden, in regressions of preparedness and COVID-19 burden. Inclusion of average stringency index [26] as a measure of COVID-19 mitigation was also done in another study assessing preparedness on COVID-19 burden [27].

## Results

### Descriptive statistics

The 2021 GHS Index ranges from 16.0 to 75.9 with a global population-weighted average score of 45.2 (Figure S1). When stratified by IHME super-regions [28], High-income super-region had the highest GHS Index score at 65.8 followed by Latin America and Caribbean (47.9), Southeast Asia, East Asia, and Oceania (47.3), Central Europe, Eastern Europe, and Central Asia (46.7), South Asia (40.5), North Africa and Middle East (33.1), and Sub-Saharan Africa (32.9) (Table S2).

**Fig. 1:**
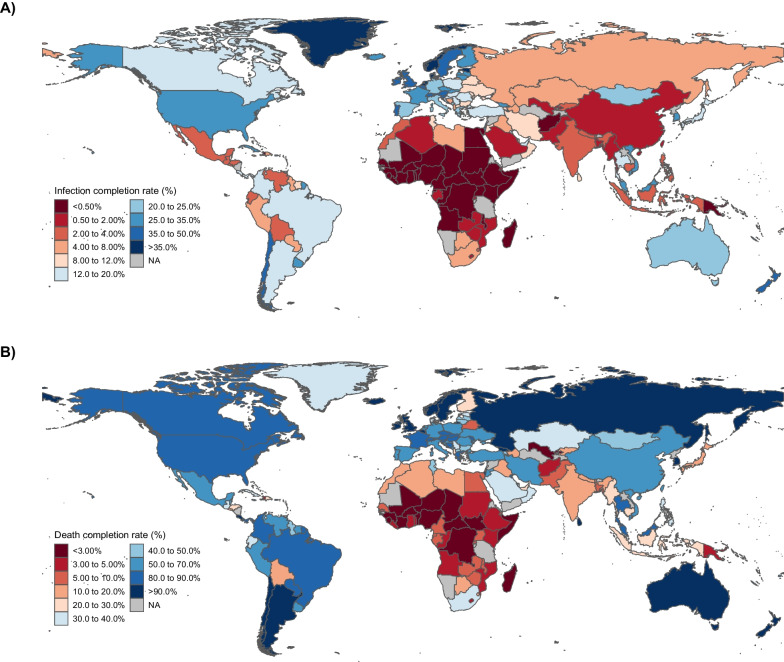
Global distribution of cumulative SARS-CoV-2 **A** infection completion rates and **B** COVID-19 death completion rates, 2020–2021. Caption: Country-level infection completion rates computed by dividing cumulative reported cases by cumulative SARS-CoV-2 infections up to the end of 2021. Country-level death completion rates computed by dividing cumulative reported deaths by cumulative deaths where individuals were actively infected with COVID-19 at the time of death up to the end of 2021

Country-level infection completion rates ranged from 0.02 to 68.1% with a global rate of 5.32% during the period from the beginning of the pandemic to the end of 2021 (Fig. 1). The High-income super-region had the largest completion rate with 28.2% while Sub-Saharan Africa had the lowest at 0.45% (Table S2). At the global-level, completion rates varied as the pandemic progressed as the completion rate was at 2.67% 100 days after the first reported case in each country to 6.68% in the 300–500 days period but decreased in the final observed period to 4.43% at 500–700 days (Table S3). Country-level death completion rates ranged from 0.85% to 100.0% with a global completion rate of 38.7% in the period representing the beginning of the pandemic to the end of 2021 (Fig. 1). Similar to infection completion rates, Sub-Saharan Africa had the lowest death completion rate at 8.81% while the High-income region had the highest at 74.6% (Table S2).

### Relationship between pandemic preparedness capacities and SARS-CoV-2 infection completion rates

After adjustment for GDP per capita, the GHS Index was positively associated with SARS-CoV-2 infection completion rates and the effect size increased as the pandemic progressed (Table 1), increasing from 1.09 (95% CI 1.04–1.16) at 100 days to 1.17 (1.10–1.25) at the 500–700-day period. During the entire cumulative 700-day observational period, each 10% increase in the GHS Index was associated with a 14.9% (8.34–21.8%) increase in the SARS-CoV-2 infection completion rate.

**Table 1:** Country-level effect sizes of pandemic preparedness capacities on SARS-CoV-2 infection completion rates stratified by time periods after the first reported case in each country

Pandemic preparedness capacity	SARS-CoV-2 infection completion rate ratios					
	Days ≤ 100	Days 100–300	Day 300–500	Days 500–700	Total observation period	Ratio
*Global health security index score*	1.09 (1.04, 1.16)	1.14 (1.07, 1.22)	1.11 (1.04, 1.19)	1.17 (1.10, 1.25)	1.15 (1.08, 1.22)	1.06 (1.00, 1.13)
Prevention score	1.05 (1.03, 1.07)	1.07 (1.05, 1.09)	1.07 (1.04, 1.10)	1.08 (1.06, 1.11)	1.08 (1.05, 1.10)	1.03 (1.01, 1.05)
Zoonotic disease	1.02 (1.00, 1.03)	1.02 (1.01, 1.04)	1.03 (1.01, 1.04)	1.03 (1.01, 1.05)	1.03 (1.01, 1.04)	1.01 (1.00, 1.02)
Detection score	1.03 (1.01, 1.05)	1.04 (1.01, 1.07)	1.02 (1.00, 1.05)	1.04 (1.02, 1.07)	1.04 (1.01, 1.06)	1.02 (0.99, 1.04)
Laboratory capacity						
Low capacity	1.30 (0.80, 2.12)	1.22 (0.76, 1.96)	1.08 (0.64, 1.82)	1.11 (0.66, 1.85)	1.11 (0.69, 1.79)	0.83 (0.54, 1.28)
Moderate capacity	1.18 (0.76, 1.81)	1.66 (1.05, 2.62)	1.38 (0.83, 2.28)	1.21 (0.71, 2.09)	1.32 (0.81, 2.15)	1.05 (0.66, 1.67)
Moderate-high capacity	1.81 (1.20, 2.74)	2.01 (1.31, 3.08)	1.79 (1.11, 2.89)	2.26 (1.40, 3.63)	2.11 (1.37, 3.25)	1.23 (0.82, 1.85)
Laboratory quality systems						
Moderate quality	1.05 (0.66, 1.67)	0.86 (0.53, 1.42)	0.66 (0.39, 1.12)	0.71 (0.41, 1.22)	0.70 (0.42, 1.15)	0.71 (0.45, 1.14)
High quality	1.53 (1.07, 2.19)	1.68 (1.12, 2.51)	1.46 (0.96, 2.21)	1.91 (1.23, 2.95)	1.65 (1.11, 2.45)	1.26 (0.84, 1.88)
Case-based investigation	1.01 (1.00, 1.02)	1.01 (1.00, 1.02)	1.01 (1.00, 1.02)	1.01 (1.00, 1.02)	1.01 (1.00, 1.02)	1.00 (0.99, 1.01)
Laboratory supply chain						
Moderate	0.94 (0.68, 1.28)	0.96 (0.70, 1.30)	1.10 (0.74, 1.64)	0.99 (0.66, 1.49)	1.04 (0.74, 1.47)	1.06 (0.70, 1.60)
High	1.15 (0.61, 2.15)	0.75 (0.31, 1.82)	0.53 (0.09, 3.07)	2.18 (0.95, 5.01)	1.62 (0.72, 3.63)	1.90 (1.22, 2.97)
Real-time surveillance/reporting	1.00 (0.99, 1.01)	1.00 (0.99, 1.01)	0.99 (0.98, 1.01)	1.00 (0.99, 1.01)	1.00 (0.99, 1.01)	1.00 (0.99, 1.01)
Response score	1.02 (0.96, 1.08)	1.04 (0.97, 1.11)	1.04 (0.97, 1.11)	1.08 (1.00, 1.16)	1.06 (1.00, 1.13)	1.06 (0.99, 1.13)
Emergency preparedness and response planning	1.01 (0.99, 1.02)	1.01 (0.99, 1.03)	1.01 (0.99, 1.03)	1.02 (1.00, 1.04)	1.02 (1.00, 1.04)	1.01 (0.99, 1.02)
Access to communications infrastructure	1.14 (1.04, 1.24)	1.20 (1.09, 1.32)	1.22 (1.08, 1.37)	1.19 (1.08, 1.32)	1.20 (1.08, 1.33)	1.05 (0.99, 1.12)
Health system score	1.05 (1.03, 1.08)	1.07 (1.04, 1.10)	1.06 (1.03, 1.09)	1.07 (1.03, 1.11)	1.06 (1.03, 1.10)	1.01 (0.98, 1.04)
Health capacity in healthcare settings	1.02 (1.00, 1.03)	1.03 (1.01, 1.05)	1.03 (1.00, 1.05)	1.04 (1.02, 1.06)	1.03 (1.01, 1.05)	1.02 (1.00, 1.03)
Healthcare access	1.06 (1.00, 1.11)	1.08 (1.00, 1.15)	1.10 (1.02, 1.18)	1.06 (1.00, 1.13)	1.07 (1.01, 1.14)	1.01 (0.97, 1.05)
International norms score	0.99 (0.94, 1.05)	1.03 (0.97, 1.09)	0.98 (0.92, 1.04)	1.03 (0.97, 1.09)	1.01 (0.96, 1.07)	1.04 (0.98, 1.09)
Cross-border agreements on health responses						
Moderate	0.72 (0.49, 1.04)	0.86 (0.57, 1.31)	0.56 (0.36, 0.87)	0.65 (0.41, 1.05)	0.64 (0.42, 0.97)	0.88 (0.58, 1.33)
High	0.80 (0.54, 1.16)	1.15 (0.75, 1.76)	0.80 (0.50, 1.29)	1.15 (0.71, 1.84)	0.98 (0.65, 1.48)	1.41 (0.88, 2.25)
International commitments	1.03 (1.00, 1.05)	1.04 (1.01, 1.07)	1.04 (1.01, 1.07)	1.05 (1.02, 1.08)	1.05 (1.02, 1.08)	1.02 (1.00, 1.05)
Risk environment score	1.21 (1.11, 1.31)	1.24 (1.12, 1.37)	1.24 (1.11, 1.39)	1.27 (1.14, 1.41)	1.27 (1.15, 1.41)	1.04 (0.95, 1.14)
Government effectiveness	1.05 (1.02, 1.08)	1.07 (1.03, 1.11)	1.05 (1.01, 1.09)	1.07 (1.03, 1.12)	1.07 (1.03, 1.11)	1.02 (0.99, 1.05)
Socioeconomic resilience	1.16 (1.09, 1.23)	1.25 (1.18, 1.33)	1.24 (1.15, 1.33)	1.30 (1.21, 1.39)	1.28 (1.20, 1.36)	1.11 (1.04, 1.19)
Social inclusion	1.05 (1.02, 1.09)	1.08 (1.04, 1.11)	1.08 (1.03, 1.12)	1.07 (1.02, 1.13)	1.07 (1.03, 1.12)	1.02 (0.99, 1.05)
Inequality	1.00 (0.99, 1.02)	1.01 (0.99, 1.03)	1.00 (0.99, 1.02)	1.02 (1.00, 1.03)	1.01 (1.00, 1.03)	1.01 (1.00, 1.03)
Trust in health advice from government						
Moderate trust	0.99 (0.66, 1.47)	1.02 (0.68, 1.54)	0.94 (0.57, 1.54)	0.76 (0.46, 1.26)	0.91 (0.57, 1.46)	0.79 (0.58, 1.07)
High trust	1.13 (0.73, 1.75)	0.86 (0.54, 1.37)	0.64 (0.36, 1.12)	0.79 (0.46, 1.34)	0.89 (0.55, 1.45)	0.70 (0.48, 1.04)
Trust in health advice from health care worker						
Moderate trust	3.60 (1.94, 6.67)	3.37 (2.01, 5.65)	4.10 (2.32, 7.25)	3.20 (1.51, 6.81)	3.98 (2.16, 7.34)	0.92 (0.61, 1.38)
High trust	3.87 (2.04, 7.33)	4.01 (2.32, 6.92)	4.01 (2.24, 7.18)	3.82 (1.76, 8.26)	4.48 (2.38, 8.42)	1.00 (0.63, 1.59)
*Joint external evaluation (JEE) ready score*	1.17 (1.07, 1.27)	1.15 (1.07, 1.25)	1.13 (1.03, 1.24)	1.19 (1.09, 1.30)	1.19 (1.10, 1.30)	1.01 (0.93, 1.10)
Prevention score	1.17 (1.08, 1.26)	1.17 (1.08, 1.28)	1.16 (1.06, 1.26)	1.22 (1.11, 1.33)	1.21 (1.12, 1.32)	1.03 (0.95, 1.12)
Detection score	1.14 (1.00, 1.29)	1.15 (1.03, 1.29)	1.12 (0.98, 1.28)	1.25 (1.11, 1.42)	1.23 (1.09, 1.39)	1.06 (0.94, 1.21)
Response score	1.10 (1.02, 1.18)	1.07 (1.00, 1.14)	1.05 (0.97, 1.13)	1.08 (1.00, 1.16)	1.08 (1.01, 1.16)	0.97 (0.91, 1.03)

Effect sizes are rate ratios comparing 10% differences in each index. Individual regressions were implemented for each GHS Index measure to assess the effect of the measure independent of other indicators. Covariates included in each regression was log transformed pre-pandemic gross domestic product (GDP) per capita. The ratio column reports effect sizes where the ratio of SARS-CoV-2 infection completion rates in the 500–700 day period compared to days 0–100 was the outcome variable. One was added to continuous preparedness indicators prior to log transformation

Each of the six GHS Index categories were positively associated with infection completion rates with the exception of the international norms category during the entire observation period. The effect sizes for prevention, detection, response, health system, international norms, and risk environment capacities were the following at 700 days: 1.08 (1.05–1.10), 1.04 (1.01–1.06), 1.06 (1.00–1.13), 1.06 (1.03–1.10), 1.01 (0.96, 1.07), and 1.27 (1.15, 1.41), respectively. The effect sizes for each of these categories generally increased as the pandemic progressed.

Several GHS Index indicators were positively associated with SARS-CoV-2 infection completion rates. Laboratory capacity (Moderate-high vs None: β = 2.11 [1.37–3.25]), Laboratory quality (High vs None: β = 1.65 [1.11–2.45]), case-based investigation tools (β = 1.01 [1.003–1.02]) in regards to detection capacities were associated with infection completion. For response capacities, emergency preparedness planning (β = 1.02 [1.00–1.04]) and communication infrastructure (β = 1.20 [1.08–1.33]) remained related to infection completion rates. Health capacity in healthcare settings (β = 1.03 [1.01–1.05]) and healthcare access (β = 1.07 [1.01–1.14]) were health system capacities that were positively associated with infection completion rates. Although the international norms category was not related to infection detection, the international commitments capacity (β = 1.05 [1.02, 1.08]) were positively associated with infection completion.

The risk environment category had the largest effect size for the six categories such that each 5-point increase in the risk environment was associated with a 27.0% (14.5–40.9%) increase in SARS-CoV-2 completion rate. Most risk environment indicators were associated with infection completion including government effectiveness (β = 1.07 [1.03–1.11]), socio-economic resilience (β = 1.28 [1.20–1.36]), social inclusion (β = 1.07 [1.03–1.12]), and trust in health advice from health care workers (High vs None: β = 4.48 [2.38–8.42]).

The GHS Index was also positively associated with the ratio of SARS-CoV-2 infection completions comparing completion rates at the 500-to-700-day period to day 100 (β = 1.06 [1.00–1.13]), indicating that each 5-point increase in the GHS Index was associated with a 6.23% (− 0.17 to 13.0%) increase in the completion rate ratio. Most individual GHS Index categories were also associated with SARS-CoV-2 infection completion rates.

### Pandemic preparedness capacities and COVID-19 death completion rates

After adjustment for income, the GHS Index was positively associated with COVID-19 death completion rate (β = 1.11 [1.06–1.15], Table 2), indicating that each 5-point increase in the GHS Index was associated with a 10.6% (5.91–15.4%) increase in the death completion rates. In regards to the GHS categories, the effect sizes for prevention, detection, response, health system, international norms, and risk environment capacities were the following: 1.05 (1.04–1.07), 1.03 (1.01–1.05), 1.05 (1.00–1.10), 1.05 (1.03–1.07), 1.01 (0.97–1.05), and 1.15 (1.08–1.23), respectively. Most indicators that were associated with infection completion rates remained associated with death completion rates, though the level varied. However, unlike for infection completions, the relationship remained the same throughout the pandemic for deaths (Table S4). For both data completion outcomes, results were consistent when utilizing the Joint External Evaluation (JEE) ready score as an alternative measure of preparedness with the effect size for infection completion during the observation period being 1.19 (1.10–1.30) and 1.16 (1.10–1.22) for death completion (Tables 1 and 2). We also observed positive associations when examining the prevention (infection: β = 1.21 [1.12–1.32], deaths: β = 1.16 [1.10–1.22]), detection (infection: β = 1.23 [1.09–1.39], deaths: β = 1.18 [1.10–1.28]), and response (infection: β = 1.08 [1.01–1.16], deaths: β = 1.08 [1.03–1.13]) sub-indicators of the JEE.

**Table 2:** Country-level effect sizes of pandemic preparedness capacities on cumulative COVID-19 death completion rates and ratios of COVID-19 excess mortality to reported COVID-19 mortality

Pandemic preparedness capacity	COVID-19 death completion rate ratio	Ratio of excess mortality to reported COVID-19 mortality		
		The Economist	IHME	WHO
*Global health security index score*	1.11 (1.06, 1.15)	− 0.02 (− 0.09, 0.04)	− 0.06 (− 0.10, − 0.02)	− 0.02 (− 0.08, 0.05)
Prevention score	1.05 (1.04, 1.07)	− 0.01 (− 0.03, 0.02)	− 0.03 (− 0.04, − 0.01)	− 0.00 (− 0.03, 0.02)
Zoonotic disease	1.02 (1.01, 1.03)	− 0.00 (− 0.02, 0.02)	− 0.01 (− 0.02, − 0.00)	0.00 (− 0.02, 0.02)
Detection score	1.03 (1.01, 1.05)	0.01 (− 0.02, 0.04)	− 0.02 (− 0.04, − 0.00)	− 0.01 (− 0.04, 0.03)
Laboratory capacity				
Low capacity	0.88 (0.62, 1.25)	− 0.36 (− 0.93, 0.22)	− 0.08 (− 0.41, 0.25)	− 0.39 (− 0.99, 0.21)
Moderate capacity	1.28 (0.92, 1.77)	− 0.04 (− 0.37, 0.30)	− 0.07 (− 0.37, 0.24)	− 0.04 (− 0.42, 0.33)
Moderate− high capacity	1.78 (1.29, 2.45)	− 0.47 (− 0.95, 0.01)	− 0.48 (− 0.79, − 0.16)	− 0.57 (− 1.07, − 0.07)
Laboratory quality systems				
Moderate quality	1.02 (0.71, 1.47)	0.40 (− 0.17, 0.97)	0.14 (− 0.17, 0.44)	0.42 (− 0.12, 0.96)
High quality	1.56 (1.15, 2.12)	0.15 (− 0.27, 0.58)	− 0.30 (− 0.57, − 0.02)	− 0.07 (− 0.52, 0.38)
Case-based investigation	1.01 (1.00, 1.01)	− 0.00 (− 0.01, 0.01)	− 0.01 (− 0.02, − 0.00)	− 0.01 (− 0.02, 0.00)
Laboratory supply chain				
Moderate	0.80 (0.61, 1.06)	0.21 (− 0.09, 0.52)	0.19 (− 0.06, 0.45)	0.31 (− 0.01, 0.64)
High	1.04 (0.45, 2.40)	− 0.95 (− 2.42, 0.51)	− 0.59 (− 1.96, 0.77)	− 1.17 (− 2.49, 0.14)
Real-time surveillance/reporting	1.00 (0.99, 1.01)	0.01 (− 0.00, 0.02)	0.00 (− 0.00, 0.01)	0.01 (− 0.01, 0.02)
Response score	1.05 (1.00, 1.10)	− 0.03 (− 0.10, 0.03)	− 0.03 (− 0.07, 0.01)	− 0.01 (− 0.07, 0.05)
Emergency preparedness and response planning	1.01 (1.00, 1.03)	0.00 (− 0.02, 0.03)	− 0.01 (− 0.02, 0.00)	0.01 (− 0.02, 0.03)
Access to communications infrastructure	1.16 (1.07, 1.25)	− 0.14 (− 0.22, − 0.05)	− 0.09 (− 0.15, − 0.03)	− 0.09 (− 0.18, − 0.01)
Health system score	1.05 (1.03, 1.07)	− 0.01 (− 0.04, 0.02)	− 0.03 (− 0.05, − 0.00)	0.01 (− 0.03, 0.04)
Health capacity in healthcare settings	1.02 (1.00, 1.03)	− 0.02 (− 0.04, 0.00)	− 0.01 (− 0.03, 0.00)	− 0.02 (− 0.04, 0.00)
Healthcare access	1.04 (0.98, 1.09)	− 0.02 (− 0.06, 0.02)	− 0.01 (− 0.06, 0.03)	− 0.01 (− 0.06, 0.05)
International norms score	1.01 (0.97, 1.05)	0.01 (− 0.06, 0.07)	− 0.01 (− 0.06, 0.03)	0.02 (− 0.04, 0.08)
Cross-border agreements on health responses				
Moderate	0.99 (0.72, 1.37)	0.25 (− 0.23, 0.74)	0.06 (− 0.23, 0.36)	0.25 (− 0.26, 0.76)
High	1.01 (0.72, 1.43)	0.17 (− 0.30, 0.63)	− 0.01 (− 0.34, 0.33)	0.21 (− 0.26, 0.68)
International commitments	1.03 (1.01, 1.05)	0.00 (− 0.03, 0.03)	− 0.02 (− 0.03, 0.00)	0.00 (− 0.03, 0.03)
Risk Environment score	1.15 (1.08, 1.23)	− 0.17 (− 0.28, − 0.06)	− 0.10 (− 0.17, − 0.03)	− 0.17 (− 0.28, − 0.06)
Government effectiveness	1.05 (1.02, 1.07)	− 0.05 (− 0.09, − 0.00)	− 0.03 (− 0.05, − 0.00)	− 0.05 (− 0.09, − 0.00)
Socioeconomic resilience	1.18 (1.13, 1.23)	− 0.16 (− 0.24, − 0.08)	− 0.12 (− 0.18, − 0.07)	− 0.13 (− 0.21, − 0.05)
Social inclusion	1.06 (1.03, 1.09)	− 0.04 (− 0.07, − 0.02)	− 0.04 (− 0.07, − 0.02)	− 0.02 (− 0.05, 0.01)
Inequality	1.01 (1.00, 1.02)	− 0.01 (− 0.02, 0.01)	− 0.01 (− 0.02, 0.00)	− 0.01 (− 0.02, 0.01)
Trust in health advice from government				
Moderate trust	1.01 (0.70, 1.47)	− 0.36 (− 0.75, 0.04)	− 0.04 (− 0.34, 0.25)	− 0.28 (− 0.82, 0.27)
High trust	1.05 (0.70, 1.57)	− 0.07 (− 0.52, 0.38)	− 0.12 (− 0.54, 0.30)	− 0.13 (− 0.75, 0.48)
Trust in health advice from health care worker				
Moderate trust	3.12 (2.29, 4.26)	− 1.28 (− 2.00, − 0.55)	− 0.74 (− 1.18, − 0.29)	− 0.30 (− 2.21, 1.61)
High trust	2.96 (2.12, 4.13)	− 0.93 (− 1.65, − 0.21)	− 0.68 (− 1.16, − 0.20)	− 0.09 (− 1.98, 1.81)
*Joint external evaluation (JEE) ready score*	1.16 (1.10, 1.22)	− 0.02 (− 0.11, 0.06)	− 0.08 (− 0.15, − 0.01)	− 0.07 (− 0.15, 0.01)
Prevention score	1.16 (1.10, 1.22)	− 0.06 (− 0.13, 0.01)	− 0.09 (− 0.15, − 0.03)	− 0.12 (− 0.19, − 0.04)
Detection score	1.18 (1.10, 1.28)	0.01 (− 0.11, 0.12)	− 0.11 (− 0.20, − 0.03)	− 0.08 (− 0.18, 0.01)
Response score	1.08 (1.03, 1.13)	− 0.00 (− 0.09, 0.09)	− 0.04 (− 0.09, 0.01)	− 0.01 (− 0.09, 0.07)

Effect sizes are rate ratios comparing 10% differences in each index. Individual regressions were implemented for each GHS Index measure to assess the effect of the measure independent of other indicators. Covariates included in each regression was log transformed pre-pandemic gross domestic product (GDP) per capita. Ratio of excess mortality to reported COVID-19 mortality were log-modulus transformed prior to regressions owing to potential negative excess deaths for some countries. One was added to continuous preparedness indicators prior to log transformation

### Pandemic preparedness-COVID burden relationship adjusted for differences in completion rates

**Fig. 2:**
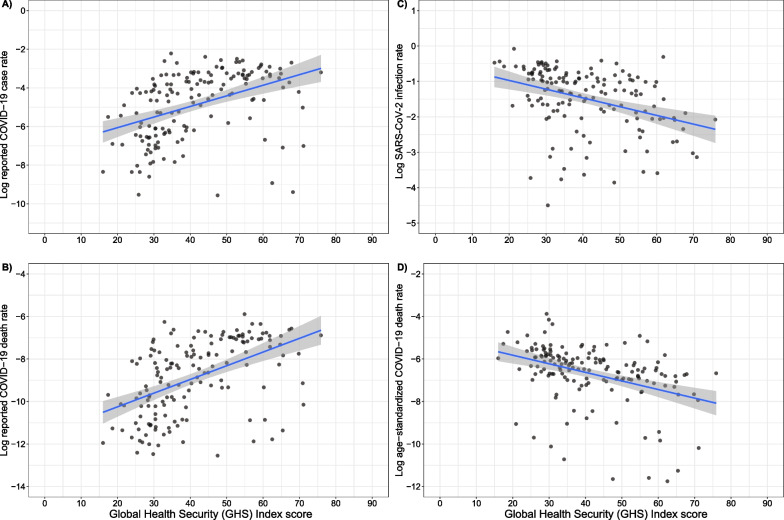
Relationships between the Global Health Security (GHS) Index and SARS-CoV-2 infection and death rates before and after adjustment for differences in detection during the pre-vaccine era. Caption: The infection rate panels illustrate cumulative infection rates after 300 days of the first reported case in each country. The death panels illustrate cumulative deaths rates after 300 days of the first reported death in each country. Age-standardization in panel D was conducting utilizing indirect standardization with the comparative mortality ratio approach. The blue line is a linear regression line while the shaded area is the corresponding 95% confidence interval

After adjustment for completion rates, the bivariate relationship between the GHS Index on SARS-CoV-2 infection and mortality rates reverses direction, compared to when reported rates are used, such that they are in the negative direction (Fig. 2). For example, the relationship between the GHS Index and reported COVID-19 cases was null at 100 days (β = 0.95 [0.85–1.05]) and at 300 days (β = 1.02 [0.92–1.14]) after the first reported case when holding log-pre-pandemic income constant (Table 3). However, when SARS-CoV-2 infection rate was the outcome, each 10% increase in the GHS Index was associated with a 13.5% (4.80–21.4%) decrease in infection rates at 100 days and a 9.10 (1.07–16.5%) decrease at 300 days. Most capacities remained negatively associated with SARS-CoV-2 infection rates including prevention (β = 0.97 [0.95–1.00]), detection (β = 0.96 [0.93–0.99]), international norms (β = 0.93 [0.87–1.00]), risk environment (β = 0.87 [0.80–0.93]). The largest effect sizes were for the JEE (β = 0.84 [0.76–0.94]) and the JEE sub-indicators (Table 3).

**Table 3:** Country-level effect sizes of pandemic preparedness capacities on cumulative reported COVID-19 case rates and SARS-CoV-2 infection rates at 100 and 300 days after first reported case for each country

Pandemic preparedness capacity	COVID-19 case rate ratios		SARS-CoV-2 infection rate ratios	
	Day 100	Day 300	Day 100	Day 300
Global health security index score	0.95 (0.85, 1.05)	1.02 (0.92, 1.14)	0.86 (0.79, 0.95)	0.91 (0.84, 0.99)
Prevention score	0.99 (0.95, 1.03)	1.04 (1.00, 1.07)	0.94 (0.91, 0.97)	0.97 (0.95, 1.00)
Detection score	0.98 (0.94, 1.01)	1.00 (0.95, 1.04)	0.95 (0.92, 0.98)	0.96 (0.93, 0.99)
Response score	0.94 (0.85, 1.03)	0.96 (0.87, 1.05)	0.92 (0.84, 1.02)	0.93 (0.86, 1.01)
Health system score	1.02 (0.98, 1.07)	1.06 (1.01, 1.11)	0.97 (0.93, 1.01)	0.99 (0.96, 1.03)
International norms score	0.90 (0.82, 0.98)	0.95 (0.87, 1.04)	0.91 (0.84, 0.98)	0.93 (0.87, 1.00)
Risk environment score	0.97 (0.86, 1.11)	1.05 (0.94, 1.18)	0.81 (0.71, 0.91)	0.86 (0.80, 0.93)
Joint external evaluation (JEE) ready score	0.90 (0.77, 1.06)	0.98 (0.85, 1.12)	0.77 (0.68, 0.88)	0.84 (0.76, 0.94)
Prevention score	0.88 (0.76, 1.03)	0.98 (0.86, 1.13)	0.76 (0.67, 0.86)	0.83 (0.74, 0.93)
Detection score	0.82 (0.66, 1.00)	0.90 (0.75, 1.08)	0.72 (0.62, 0.83)	0.78 (0.69, 0.89)
Response score	0.98 (0.88, 1.09)	1.00 (0.92, 1.09)	0.89 (0.82, 0.97)	0.93 (0.88, 0.99)

Effect sizes are rate ratios comparing 10% differences in each index. Individual regressions were implemented for each GHS Index measure to assess the effect of the measure independent of other indicators. Covariates included in each regression was log transformed pre-pandemic gross domestic product (GDP) per capita. One was added to continuous preparedness indicators prior to log transformation

There was a similar change in relationships when examining COVID-19 death rates (Table 4). When reported COVID-19 death rates was the outcome, the relationship between the GHS Index and reported COVID-19 deaths trended in the positive direction at 300 days (β = 1.03 [0.93–1.14]) holding log-pre-pandemic income constant. After adjusting COVID-19 death rates for data completion but prior to age-standardization, the relationship was null at 300 days (β = 0.93 [0.83–1.04]). When adjusted for both data completion and age-structure, we found a relationship in the negative direction (β = 0.90 [0.81–1.00]) with every 10% in the GHS Index associated with a 10.3% (− 0.00 to 19.5%) decrease in COVID-19 death rates. Similar to infection rates, the effect of the GHS Index on age-standardized total COVID-19 death rate was largest at 100 days after the first reported case (β = 0.84 [0.75–0.95]). The effect sizes were strongest for the JEE ready score (β = 0.85 [0.75–0.97]), JEE prevention score (β = 0.84 [0.72–0.97]), and JEE detection score (β = 0.73 [0.63–0.86]).

**Table 4:** Country-level effect sizes of pandemic preparedness capacities on cumulative reported COVID-19 death rates, total COVID-19 death rates, and age-standardized total COVID-19 death rates at 100 and 300 days after first reported case for each country

Pandemic preparedness capacity	Reported COVID-19 death rate ratios		Total COVID-19 death rate ratios		Age-standardized COVID-19 death rate ratios	
	Day 100	Day 300	Day 100	Day 300	Day 100	Day 300
Global health security index score	1.03 (0.93, 1.14)	1.09 (0.98, 1.22)	0.93 (0.83, 1.04)	0.99 (0.89, 1.10)	0.84 (0.75, 0.95)	0.90 (0.81, 1.00)
Prevention score	1.02 (0.98, 1.06)	1.06 (1.02, 1.11)	0.97 (0.93, 1.01)	1.01 (0.98, 1.05)	0.93 (0.90, 0.97)	0.97 (0.94, 1.00)
Detection score	0.99 (0.95, 1.03)	1.01 (0.97, 1.05)	0.96 (0.92, 1.00)	0.98 (0.94, 1.02)	0.94 (0.90, 0.98)	0.96 (0.92, 1.00)
Response score	1.00 (0.91, 1.11)	1.00 (0.89, 1.11)	0.96 (0.86, 1.08)	0.95 (0.86, 1.06)	0.92 (0.81, 1.04)	0.91 (0.81, 1.01)
Health system score	1.04 (0.99, 1.09)	1.08 (1.04, 1.13)	0.99 (0.94, 1.04)	1.03 (0.99, 1.08)	0.95 (0.90, 1.00)	0.99 (0.95, 1.04)
International norms score	0.99 (0.91, 1.09)	1.00 (0.92, 1.09)	0.99 (0.89, 1.09)	0.99 (0.91, 1.08)	0.93 (0.84, 1.03)	0.94 (0.86, 1.02)
Risk environment score	0.90 (0.79, 1.03)	1.06 (0.94, 1.19)	0.78 (0.67, 0.91)	0.92 (0.83, 1.01)	0.72 (0.61, 0.84)	0.84 (0.76, 0.93)
Joint external evaluation (JEE) ready score	1.01 (0.87, 1.17)	1.06 (0.93, 1.21)	0.87 (0.75, 1.02)	0.92 (0.81, 1.05)	0.81 (0.69, 0.95)	0.85 (0.75, 0.97)
Prevention score	0.98 (0.85, 1.12)	1.05 (0.91, 1.20)	0.84 (0.73, 0.97)	0.91 (0.79, 1.04)	0.78 (0.67, 0.90)	0.84 (0.72, 0.97)
Detection score	0.93 (0.77, 1.12)	0.96 (0.80, 1.15)	0.78 (0.64, 0.95)	0.81 (0.69, 0.95)	0.71 (0.58, 0.86)	0.73 (0.63, 0.86)
Response score	1.05 (0.95, 1.16)	1.07 (0.98, 1.15)	0.97 (0.87, 1.08)	0.99 (0.92, 1.06)	0.95 (0.85, 1.06)	0.96 (0.89, 1.03)

Effect sizes are rate ratios comparing 10% differences in each index. Individual regressions were implemented for each GHS Index measure to assess the effect of the measure independent of other indicators. Covariates included in each regression was log transformed pre-pandemic gross domestic product (GDP) per capita. One was added to continuous preparedness indicators prior to log transformation

### One-way sensitivity analyses

When utilizing COVID-19 testing rates as an alternative measure of infection completion, there was no association for the GHS Index but there were associations present for some categories (e.g., prevention: β = 1.03 [1.01–1.05], health system: β = 1.02 [0.99–1.06], risk environment: β = 1.26 [1.14–1.40], JEE response: β = 1.08 [1.00–1.17]) (Table S5). The results were mostly robust to time cutoffs as the effect sizes for infection completion rates were largely consistent to when analyses were stratified by calendar days separated by six months (Tables S6 and S7). However, effect sizes were largest at Day 100 after the first global case (Table S6). The sensitivity analyses on mortality completion rates also demonstrated similar results, as the findings remained consistent when assessing the ratio of excess mortality to reported COVID-19 mortality as an alternative measure. However, there were only associations for the GHS Index when using the IHME dataset (β = − 0.06 [− 0.10 to − 0.02]) but not for the WHO (β = − 0.02 [− 0.08–0.05]) nor The Economist (β = − 0.01 [− 0.03–0.02]; Table 2). The results for the primary analyses on completion rates did not change when utilizing the 2019 iteration of the GHS Index compared to the 2021 version (Table S8). Adjustment for COVID-19 mitigation strategies did not change results in the secondary analysis of the GHS Index on COVID-19 burden outcomes (Table S9).

### Income subgroup analyses

In decomposing the effect of income on the analyses by stratifying analyses by World Bank income groups, the effect sizes were generally the largest for the Lower Middle Income and Low Income groups. The effect size for GHS Index on infection completion during the entire observation period was 1.18 (1.04–1.34) in the Low Income group, 1.41 (1.16–1.71) in the Lower Middle Income group, 1.13 (1.04–1.23) in the Upper Middle Income group, 1.13 (1.05–1.21) in the High Income group (Table S9). The corresponding effect sizes for the GHS Index on death completion rates were 1.19 (1.09–1.31) in Low Income, 1.25 (1.10–1.43) in Lower Middle Income, 1.14 (1.08–1.20) in Upper Middle Income, and 1.02 (0.97–1.07) in High Income (Table S9).

There were similar patterns when stratifying results for SARS-CoV-2 infection rate and age-standardized COVID-19 death rates. The effect size for GHS Index on SARS-CoV-2 infection rate during the pre-vaccine era was 0.89 (0.83–0.94) in the Low Income group, 0.81 (0.64–1.01) in the Lower Middle Income group, 0.94 (0.81–1.11) in the Upper Middle Income group, 0.86 (0.75–0.98) in the High Income group (Table S10). For GHS Index on age-standardized COVID-19 death rates in the same period, the effect was 0.90 (0.80–1.01) in the Low Income group, 0.78 (0.60–1.01) in the Lower Middle Income group, 0.91 (0.74–1.12) in the Upper Middle Income group, 0.87 (0.75–1.01) in the High Income group (Table S11). For both outcomes, the effect sizes were largest at 100 days after the first reported case.

## Discussion

We observed that global levels of SARS-CoV-2 infection and mortality data were variably incomplete, findings that have important implications for understanding COVID-19 and its differential impacts on country. Our results confirm that having greater levels of pandemic preparedness capacities are associated with improved SARS-CoV-2 infection and mortality detection. In the case of infection data completeness, the strength of the pandemic preparedness and infection detection relationship increased as the pandemic progresses. These findings were consistent across inventories (GHS Index and JEE) of preparedness capacities.

Our results indicate, first, that the core preparedness capacities of disease prevention, detection, and response are associated with improved completeness of SARS-CoV-2 infection and death data. For example, we found that detection capacities such as laboratory strength and quality systems were each associated with improved COVID-19 data completeness. These findings are not surprising considering that surveillance systems are a cornerstone for monitoring ill health and disease implications. In the context of the pandemic, detection capacities in low-resourced settings were constrained by limited resources, training, laboratories, and inadequate utilization of existing surveillance infrastructure throughout the pandemic [6, 29]. These differing surveillance system capacities may be a potential reason why some countries were able to implement mass testing strategies while others were only capable of implementing highly targeted strategies (e.g., testing travelers or people with severe disease) [30–32]. Available empirical data suggests that in countries with limited testing capacities, undercounting of infections by a factor of 100 [33] and undercount deaths by a factor of 10 [34, 35] are common. Prior work also demonstrates that areas that were able to leverage their laboratory capacities during the pandemic were able to improve COVID-19 data quality and pandemic outcomes [32, 36, 37].

We also observed that many other preparedness capacities had slighter larger effects on SARS-CoV-2 data completeness compared to the detection capacity. This may be because the detection metrics are not SARS-CoV-2 specific, do not capture capacities related to developing novel tests, and are not related to rapid and at-home testing capabilities. While improved detection measures are needed, it is noteworthy that detection as measured by two indicates (GHS and JEE) were associated with improved SARS-CoV-2 data completeness. In the case of the JEE, we observed that the JEE sub-indicator that had the largest effect size was the detection sub-indicator, potentially indicating that the JEE detection measures were more optimal for measuring SARS-CoV-2 detection. Besides the detection capacity, our results illustrated that other capacities may have further facilitated improved SARS-CoV-2 data completeness. For example, we observed that response capacities, specifically capacities related to emergency preparedness planning and access to communication infrastructure, were associated with greater burden detection. The finding for emergency preparedness planning may have arisen due to previous studies demonstrating that a lack of emergency planning may lead to ineffective responses [38–40]. Having a framework for emergency response may therefore provide countries with tools to efficiently implement diagnostic programs. We also found a strong relationship between communication infrastructure and SARS-CoV-2 data completeness. The large effect size for this indicator may be owing to the importance of communication for the implementation of testing strategies as communications of disease risks has previously been shown to improve health literacy [41], uptake of preventive behaviors [42], and COVID-19 test seeking [43].

The observed health system capacities were also found to be associated with higher levels of COVID-19 data completeness. For example, the finding for healthcare access on infection completion reinforces previous work illustrating that a lack of healthcare access is a primary barrier to COVID-19 diagnostic services [44, 45]. We may have also found a relationship for capacity in healthcare setting, an indicator assessing available human resources and hospital beds, as some work indicates that a limited healthcare workforce and disjointed infrastructure was another substantial barrier to COVID-19 diagnostic services in Africa [46]. In regards to the international norms category, while the category was not associated with either infection nor death data completeness, the international commitments indicators was associated with each outcome. This provides some evidence that cross-country collaborations can be beneficial, with the EU being an example as EU countries shared COVID-19 diagnostic equipment [47] and made international commitments to provide widespread access to diagnostic services within their borders [48].

The global health security category that had the strongest effect on SARS-CoV-2 data completeness was the risk environment, which encompasses indicators evaluating the socioeconomic, political, and regulatory factors that give rise to disease outbreaks. We found that government effectiveness, an indicator capturing countries’ ability to efficiently formulate and implement policies, was positively associated with data completeness. This is consistent with work illustrating that government effectiveness is associated with improved pandemic outcomes [49], as strong leadership in the context of SARS-CoV-2 data completeness may support rapid dissemination of diagnostic supplies and testing strategies [50]. We also found that trust was strongly associated with data completeness. Other studies have also highlighted that greater trust yields lower levels of COVID-19 burden [51] because trust increases adherence to government-mandated interventions [52]. In the context of this study, the finding that trust was associated with improved data completeness may have risen owing to populations who exhibit higher levels of trust being more willing to seek diagnostic services. Interestingly, our results indicate that trust in government was not a predictor of improved completeness but trust in healthcare workers was. This is in alignment with a recent scoping review indicating that a lack of trust in healthcare workers is a substantial predictor of COVID-19 testing hesitancy [43].

Finally, these results support the hypothesis that countries with greater global health security and pandemic preparedness capacities have larger SARS-CoV-2 infection and mortality data completeness rates and lower COVID-19 disease burdens. Previous reports of a positive correlation between preparedness scores and COVID-19 outcomes may have reflected differential outcome measurement error, a bias where measurement error of the outcome differs with respect to exposure status–in early analyses of pandemic preparedness on crude measures of reported case and death rates. This bias has previously been described as a major challenge in COVID-19 epidemiologic studies [53] because it can lead to spurious relationships [54]. Some studies evaluating the contribution of preparedness capacities have attempted to account for differential measurement error by adjusting for COVID-19 testing rates [11, 14] but simulations have demonstrated that controlling for predictors of measurement error may amplify net-bias [55]. Indeed, our secondary analysis where we adjust for differentials in SARS-CoV-2 data completeness supports the pre-pandemic hypothesis that investing in pandemic preparedness capacities are associated with lower levels of SARS-CoV-2 infection and mortality. This is in alignment with a previous study that observed that global health security was associated with diminished excess mortality associated with the COVID-19 pandemic [27]. Thus, the previously reported positive relationship between pandemic preparedness and reported COVID-19 outcomes may potentially be owing to countries with stronger public health systems reporting more cases and deaths compared to countries with weaker public health systems.

Together, our analyses provide additional evidence that efforts to prepare for and respond to pandemics before they occur may be effective in limiting the burden of disease during health emergencies. The measurement error limitations in prior studies therefore underscore the need for more high-quality global data to accurately evaluate the contributions of global health security and pandemic preparedness. Interestingly, our results illustrated that the preparedness-SARS-CoV-2 burden relationship was strongest early during the pandemic (within the first 100 days of the first reported case). This may be because having existing pandemic preparedness capacities potentially allows countries to quickly mobilize existing resources to limit the initial spread of the disease. However, as the pandemic progresses, continued high adherence to mitigation measures, effective utilization of preparedness capacities, vaccination, and trust may become more important factors in relation to COVID-19 outcomes across countries.

### Strengths and limitations

This study has several strengths including the ability, for the first time, to investigate cross-country variation in SARS-CoV-2 infection and mortality data completeness and their relationship to pandemic preparedness at various timepoints in the pandemic. We also included numerous sensitivity analyses utilizing additional data sources and several measures of data completeness. Finally, this study was able to evaluate the role of a myriad of capacities in improving burden detection. This may help in prioritizing capacities to further scrutinize as unprecedented increases in development assistance towards pandemic preparedness in low- to middle-income countries (LMICs) [56] are distributed. Our results illustrate that LMICs may particularly benefit from improving their preparedness capacities as our sub-analyses suggested that the effect sizes for the preparedness-data completeness relationships are 2- to 4 fold greater in LMICs compared to high income countries. The same sub-analyses also illustrated that the effect sizes for pandemic preparedness on SARS-CoV-2 burden were largest in LMICs.

However, our results from this investigation should be interpreted in the context of the following limitations. First, the denominators of our completion metrics, cumulative SARS-CoV-2 infections and total COVID-19 mortality, are subject to measurement error due to varying global-levels of reliable surveillance and vital registration systems. Both data completion outcomes are therefore only best estimates as they are dependent on modeling with predictive covariates where data are sparse. Due to these data limitations, we conducted several sensitivity analyses using COVID-19 testing rates and various other sources of estimated COVID-19 mortality. While some of our sensitivity analyses confirmed our findings, using other sources of COVID-19 mortality changed results. This reinforces an urgent need of high-quality COVID-19 outcome data based on direct measurement to more accurately assess the impact of country-specific pandemic preparedness and response policies.

Second, there is also potential measurement error in the GHS Index. The measures were constructed using data that were publicly available and therefore may not capture capacities that are not published. However, utilizing the JEE index, where countries actively provide data, as a measure of preparedness yielded similar results. Third, we could not age-standardize cumulative infection rates in our secondary analysis due to a lack of high-quality age-specific infection data. Fourth, we could not assess potential effect measure modification by COVID-19 risk factors in our analysis of pandemic preparedness on COVID-19 deaths. COVID-19 risk factors (e.g., smoking prevalence, diabetes, obesity, etc.) are unlikely to be confounding variables in this analysis because although they are associated with COVID-19 deaths, they are unlikely to be causally associated with pandemic preparedness capacities. Considering the strong relationship between age and income on COVID-19 risk factors, age-standardization and income stratified analyses likely accounted for a great deal of variation in COVID-19 risk factors. Fifth, utilizing the 2021 iteration of the GHS Index limits temporality in our analysis as this GHS Index iteration includes capacities during the COVID-19 pandemic. However, our results were largely consistent when using the 2019 iteration of the GHS Index and the JEE, which captured pre-pandemic capacities. Sixth, the use of heteroscedastic robust standard errors generally led to more conservative estimates. For example, the coefficient for GHS Index-age-standardized COVID-19 death relationship at 300 days was 0.89 (0.79, 1.00) using robust standard errors but 0.89 (0.81, 0.98) when using non-robust standard errors. Finally, this is an ecologic analysis and we thus cannot make inferences at the individual-level.

## Conclusion

Countries with stronger levels of global health security and pandemic preparedness were more equipped to have successful responses as we found that preparedness capacities were associated with greater SARS-CoV-2 infection and mortality data completeness. With unprecedented amounts of development assistance being allocated to pandemic preparedness in LMICs, countries may consider further examining how global health security can improve their surveillance data completeness, representativeness, and quality. The differential capability to track COVID-19 cases and deaths across countries as a function of pandemic preparedness levels likely contributed to prior assessments of interventions on supporting effective COVID-19 responses that were potentially limited by differential outcome measurement error. Additional research is needed to accurately assess the effects of pandemic preparedness on COVID-19 outcomes as more high-quality outcome data are disseminated.

### Supplementary Information

Supplementary Material 1.

## Data Availability

Data used as input into these analyses are publicly available at https://www.ghsindex.org/report-model/ for global health security indices, https://dc-covid.site.ined.fr/en/ for age-specific COVID death counts, https://ghdx.healthdata.org/record/ihme-data/covid_19_excess_mortality for IHME excess mortality estimates, https://www.who.int/data/sets/global-excess-deaths-associated-with-covid-19-modelled-estimates for WHO excess deaths, and https://github.com/TheEconomist/covid-19-excess-deaths-tracker for The Economist excess deaths.

## Abbreviations

COVID-19: Coronavirus disease 2019
GHS: Global health security
SARS-CoV-2: Severe Acute Respiratory Syndrome Coronavirus 2
IHME: Institute for Health Metrics and Evaluation
JEE: Joint external evaluation
WHO: World Health Organization
GDP: Gross domestic product
EU: European Union
LMIC: Low- to middle-income countries
