# Supplementary material for: Pandemic preparedness improves national-level SARS-CoV-2 infection and mortality data completeness: a cross-country ecologic analysis
Source: Popul Health Metr. 2024 Jun 15;22:12. doi: 10.1186/s12963-024-00333-1 (PMC11179302; doi:10.1186/s12963-024-00333-1)
Supplement: Supplementary file 1 — Supplementary Material 1. [file 12963_2024_333_MOESM1_ESM.docx]

Supplementary Appendix to “Pandemic preparedness improves national-level COVID-19 infection and mortality detection: a cross-country ecologic analysis”

**Authors:** Jorge R. Ledesma, Irene Papanicolas, Michael A. Stoto, Stavroula A. Chrysanthopoulou, Christopher R. Isaac, Mark N. Lurie, Jennifer B. Nuzzo

Table of Contents

**Details of the Global Health Security (GHS) index** **2**

**Table. Global Health Security (GHS) Index categories, definitions, and selected indicators3**

**Methods for indirect age-standardization of COVID-19 mortality rates** **4**

**Figure. The relationship of the Global Health Security (GHS) index on (A) reported COVID-19 death rate, (B) COVID-19 death rate adjusted for under detection, and (C) indirect age-standardization 5**

**Supplemental figures** **6**

**Figure S1. Global distribution of Global Health Security index scores in 2021 6**

**Figure S2. Relationships between the Global Health Security Index and its individual categories on cumulative SARS-CoV-2 (A) infection completion rates and (B) death completion rates7**

**Supplemental tables** **8**

**Table S1. Population-weighted averages of Global Health Security index scores and category scores by IHME super-regions** **8**

**Table S2. Global- and regional-level SARS-CoV-2 infection and death completion rates up to December 31 20218**

**Table S3. Global- and regional-level SARS-CoV-2 infection completion rates stratified by time periods after first reported case in each country 9**

**Table S4. Country-level effect sizes of pandemic preparedness capacities on COVID-19 death completion rates stratified by time periods after first reported case in each country9**

**Table S5. Country-level effect sizes of pandemic preparedness capacities on COVID-19 testing rates stratified by time periods after first reported case in each country 10**

**Table S6. Country-level effect sizes of pandemic preparedness capacities on SARS-CoV-2 infection completion rates stratified by time periods after first reported case in each country** **10**

**Table S7. Country-level effect sizes of pandemic preparedness capacities on cumulative COVID-19 infection detection rate stratified by time period11**

**Table S8. Country-level effect sizes of 2019 pandemic preparedness capacities on cumulative SARS-CoV-2 infection and death completion rates 11**

**Table S9. Country-level effect sizes of pandemic preparedness capacities on COVID-19 burden adjusted for COVID-19 response stringency 13**

**Table S10. Country-level effect sizes of pandemic preparedness capacities on cumulative SARS-CoV-2 infection and death completion rates stratified by World Bank income groups at 700 days after first reported case in each country12**

**Table S11. Country-level effect sizes of pandemic preparedness capacities on cumulative SARS-CoV-2 infection rates stratified by World Bank income groups during the pre-vaccine era 13**

**Table S12. Country-level effect sizes of pandemic preparedness capacities on cumulative age-standardized COVID-19 death rates stratified by World Bank income groups during the pre-vaccine era 14**

**Table S13. Country-level effect sizes of pandemic preparedness capacities on cumulative SARS-CoV-2 infection rates and age-standardized COVID-19 deaths at 100 and 300 days [using non-robust standard errors] 15**

**Details of the Global Health Security (GHS) index**

We collected data on country-level preparedness against infectious threats from the 2021 Global Health Security (GHS) Index. The measurement exhaustively quantifies country’s abilities or potential to carry out public health functions necessary for infectious disease outbreak prevention, detection, and response. This benchmarking tool encompasses six categories of health security using publicly available data: prevention, detection and reporting, rapid response, health system, compliance with international norms, and risk environment. For this analysis, we extracted data on these six individual categories that compromise the index. These GHS Index categories are further composed of 37 indicators and 96 sub-indicators, which are measured by 171 qualitative and quantitative questions based on publicly available data for 195 countries. Since each GHS category contains various indicators and sub-indicators, we included a set of indicators identified a priori to help identify mechanisms for improved preparedness. The indices range from 0 to 100 with lower scores indicating weaker health system capacities and higher scores suggesting stronger health system capacities. The Table below provides more information of the categories and selected indicators within each category used as input into the analyses. Further details of input data and methodology of the index have previously been described in detail.^1^

**Table.** Global Health Security (GHS) Index categories, definitions, and selected indicators

| **Category** | **Definition** | **Selected indicators** |
| --- | --- | --- |
| **Prevention** | Prevention of the emergence or release of pathogens, particularly those that may constitute a Public Health Emergency of International Concern | - Antimicrobial resistance - Zoonotic disease |
| **Detection** | Early detection and reporting of epidemics of potential international concern, which may spread beyond national or regional borders | - Laboratory systems strength and quality - Case-based investigation - Epidemiology workforce |
| **Response** | Rapid response to and mitigation of the spread of an epidemic | - Emergency preparedness and response plans - Access to communication infrastructure |
| **Health system** | Sufficient and robust health system to treat the sick and protect healthcare workers | - Health capacity in clinics, hospitals - Healthcare access |
| **Compliance with international norms** | Commitments to improving national capacity, financing plans to address gaps, and adhering to global norms | - Cross-border agreements on responses - International commitments |
| **Risk environment** | Overall risk environment and country vulnerability to biological threats | - Political and security risks - Government effectiveness - Socioeconomic resilience - Public confidence in government - Public health vulnerabilities - Trust in health advice |
| Note: Some risk environment category capacities including political and security risk, inequality, and public health vulnerabilities are reverse coded such that higher levels indicate lower risks. Full methodology of GHS index found here: https://www.ghsindex.org/wp-content/uploads/2021/11/2021_GHSindex_Methodology_FINAL.pdf | | |

**Methods for indirect age-standardization of COVID-19 mortality rates**

Since age has consistently ranked as the most important risk factor for severe illness and death from COVID-19^2^, countries with greater fractions of elderly people have increased vulnerability to severe COVID-19 disease^3–5^. Therefore, we adjusted for cross-country variation in age structure in our secondary analysis by using a comparative mortality ratio (CMR) approach. Direct age-standardization requires detailed data on COVID-19 mortality by age, which are currently unavailable for most countries. The CMR approach; however, is a form of indirect age-standardization that borrows an age structure of mortality from a reference country so that only the age distribution of the countries of interest is required. CMRs have been widely used in studies to compare mortality across countries, including in comparisons of COVID-19 outcomes^6^. We derived CMRs for each country utilizing the following formula:

$${CMR}_{c}=\frac{{Excess COVID deaths}_{c}}{\sum_{i}^{A} u_{i}^{S}*p_{i}^{c}}$$

Where *c* represents the country of interest, *u* is the COVID-19 mortality for the *S* standard country at *i* age group where *A* is the maximum age group, and *p* is the population size for *c* country at *i* age group.

We utilized the United States (US) as the reference country during computations of the CMR. Thus, we first computed age-specific cumulative mortality rates for the US using age-specific COVID-19 death counts from the demography of COVID-19 deaths database^7^ and population sizes from the UN for the corresponding age ranges. Age-specific mortality rates for the US were linked with age-specific population sizes of each country to derive expected mortality. We subsequently computed country-specific CMRs by dividing observed excess COVID-19 deaths from IHME and expected mortality. Finally, we converted country-level CMRs into indirectly age-standardized COVID-19 mortality rates by multiplying the CMR by the crude all-age COVID-19 mortality rate in the US. These age-standardized COVID-19 mortality rates were computed 100 and 300 days after the first reported death in each country to account for country variability in epidemic timelines.

The figure below illustrates the impact of accounting for cross-country variation in COVID-19 mortality detection and the impact of age-standardization on the relationship between the global health security index and COVID-19 mortality.

**Figure**. The relationship of the Global Health Security (GHS) index on (A) reported COVID-19 death rate, (B) COVID-19 death rate adjusted for under detection, and (C) indirect age-standardization.


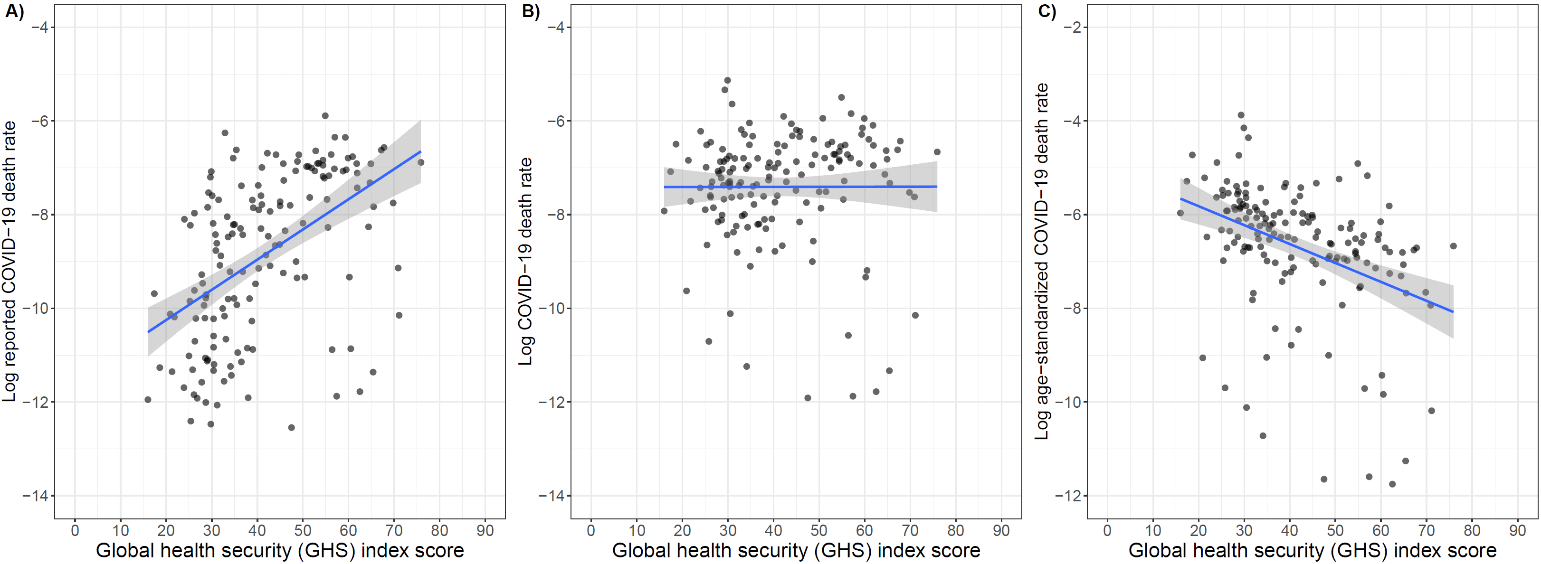


**Figure S1**. Global distribution of 2021 Global Health Security index scores.


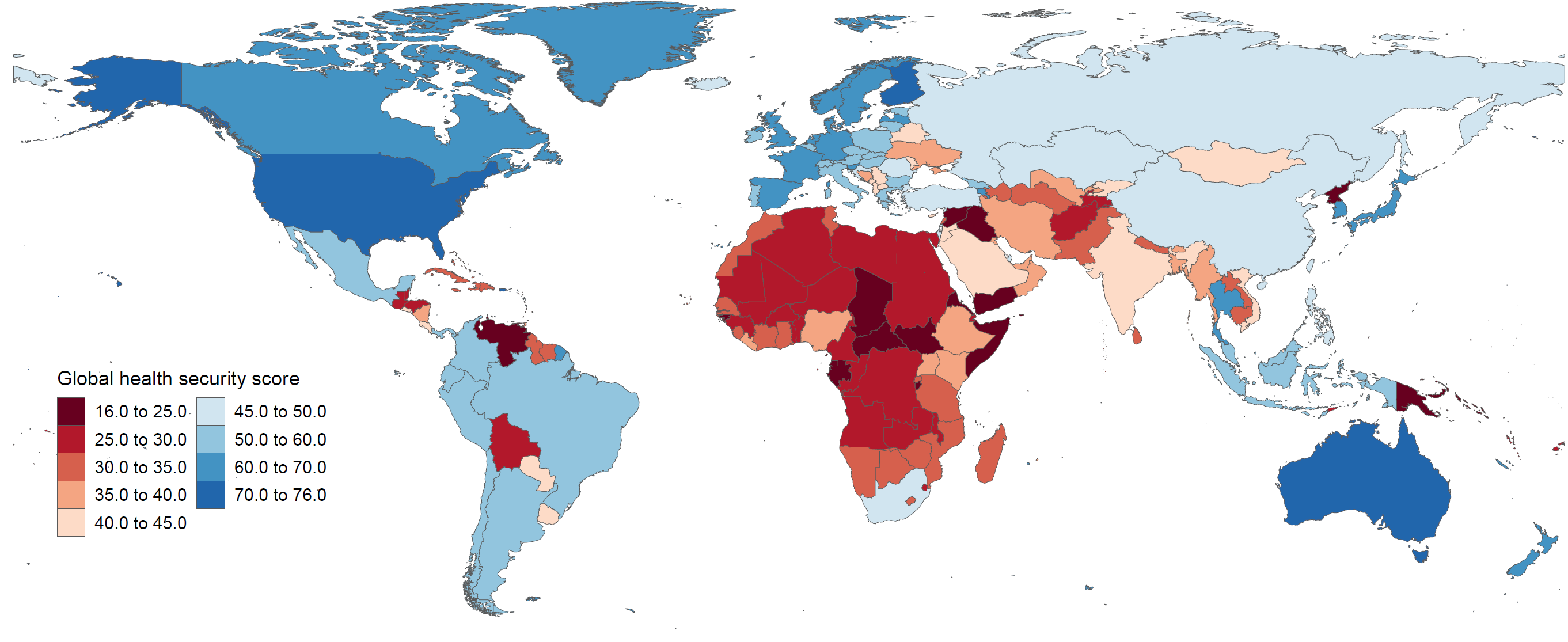


**Figure S2**. Relationships between the Global Health Security Index and its individual categories on cumulative SARS-CoV-2 (A) infection completion rates and (B) death completion rates.


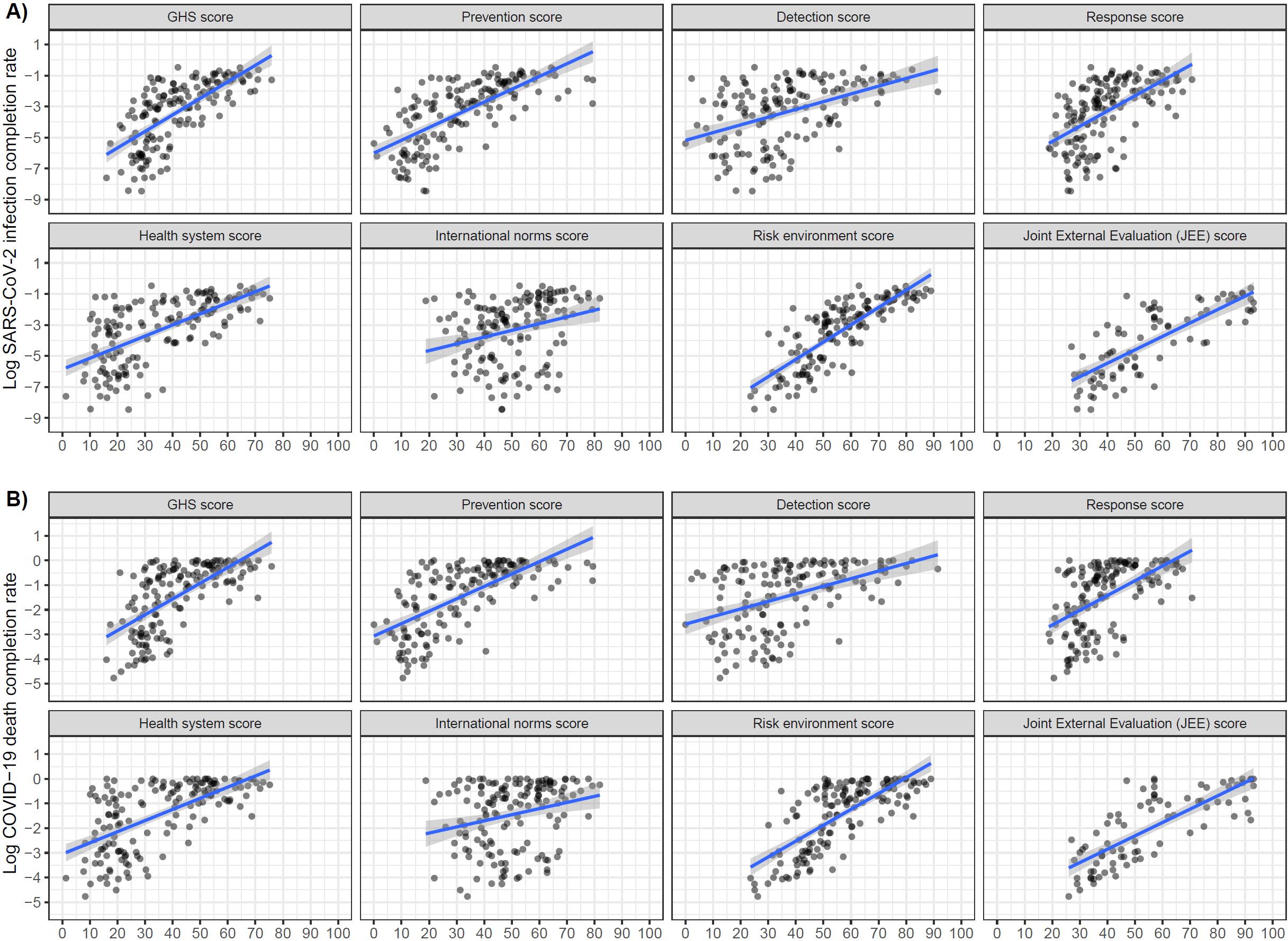


NOTE: SARS-CoV-2 infection and death completion rates computed 700 days after the first reported case in each country. The blue line is a linear regression line while the shaded area is the corresponding confidence interval.

**Table S1**. Population-weighted averages of Global Health Security index scores and category scores by IHME super-regions.

| **IHME super-region** | **GHS index score** | **Prevention score** | **Detection score** | **Response score** | **Health system score** | **International norms score** | **Risk environment score** |
| --- | --- | --- | --- | --- | --- | --- | --- |
| **Global** | **45.2** | **36.1** | **45.3** | **39.2** | **42.8** | **50.7** | **57.1** |
| Sub-Saharan Africa | 32.9 | 18.0 | 31.3 | 34.9 | 20.3 | 51.8 | 41.2 |
| North Africa and Middle East | 33.1 | 26.5 | 25.4 | 30.0 | 28.5 | 40.2 | 47.9 |
| South Asia | 40.5 | 27.6 | 41.5 | 28.7 | 41.8 | 46.3 | 57.1 |
| Central Europe, Eastern Europe, and Central Asia | 46.7 | 42.4 | 37.7 | 39.7 | 49.6 | 54.8 | 56.1 |
| Southeast Asia, East Asia, and Oceania | 47.3 | 40.2 | 50.5 | 40.1 | 46.7 | 46.0 | 60.1 |
| Latin America and Caribbean | 47.9 | 40.9 | 47.0 | 50.8 | 45.9 | 49.9 | 53.2 |
| High-income | 65.8 | 60.2 | 68.5 | 57.8 | 63.1 | 70.9 | 74.3 |
| * Global- and regional-level scores computed utilizing a population weighted average approach using county-level population sizes in 2020 | | | | | | | |

**Table S2**. Global- and regional-level SARS-CoV-2 infection and death completion rates up to December 31 2021.

| **IHME super-region** | **Infections** | **Deaths** |
| --- | --- | --- |
| **Global** | **5.32%** | **38.75%** |
| Sub-Saharan Africa | 0.45% | 8.81% |
| South Asia | 2.38% | 12.54% |
| Southeast Asia, East Asia, and Oceania | 4.02% | 30.90% |
| North Africa and Middle East | 4.96% | 25.87% |
| Latin America and Caribbean | 7.71% | 64.39% |
| Central Europe, Eastern Europe, and Central Asia | 9.37% | 67.44% |
| High-income | 28.24% | 74.57% |
| Note: Global- and regional-level metrics computed by aggregating country-level reported values and total estimated values to the regional- or global-level. | | |

**Table S3**. Global- and regional-level SARS-CoV-2 infection completion rates stratified by time periods after first reported case in each country.

| **IHME super-region** | **Day 100** | **Day 100 to Day 300** | **Day 300 to Day 500** | **Day 500 to Day 700** | **Total observation period** |
| --- | --- | --- | --- | --- | --- |
| Global | 2.67% | 4.94% | 6.68% | 4.43% | 5.15% |
| Sub-Saharan Africa | 0.33% | 0.76% | 0.77% | 0.22% | 0.41% |
| South Asia | 0.67% | 2.68% | 3.14% | 0.76% | 1.63% |
| Southeast Asia, East Asia, and Oceania | 1.33% | 2.31% | 2.90% | 3.48% | 3.14% |
| North Africa and Middle East | 2.45% | 3.97% | 6.59% | 3.03% | 3.76% |
| Latin America and Caribbean | 2.05% | 8.36% | 11.95% | 3.80% | 6.61% |
| Central Europe, Eastern Europe, and Central Asia | 3.37% | 6.60% | 11.60% | 6.39% | 7.04% |
| High-income | 14.21% | 35.13% | 35.74% | 24.94% | 28.01% |

Note: Global- and regional-level metrics computed by aggregating reported country-level values and total estimated values to the regional- or global-level.

**Table S4**. Country-level effect sizes of pandemic preparedness capacities on COVID-19 death completion rates stratified by time periods after first reported case in each country.

| **Pandemic preparedness capacity** | **COVID-19 death completion rate ratios** | | | |
| --- | --- | --- | --- | --- |
|  | **Day 100** | **Day 100 to Day 300** | **Day 300 to Day 500** | **Day 500 to Day 700** |
| ***Global Health Security Index score*** | 1.11 (1.06, 1.15) | 1.11 (1.06, 1.15) | 1.11 (1.06, 1.16) | 1.11 (1.06, 1.16) |
| **Prevention score** | 1.05 (1.04, 1.07) | 1.05 (1.04, 1.07) | 1.05 (1.04, 1.07) | 1.05 (1.04, 1.07) |
| **Detection score** | 1.03 (1.01, 1.05) | 1.03 (1.01, 1.05) | 1.03 (1.01, 1.05) | 1.03 (1.01, 1.05) |
| **Response score** | 1.05 (1.00, 1.10) | 1.05 (1.00, 1.10) | 1.05 (1.00, 1.10) | 1.05 (1.00, 1.10) |
| **Health system score** | 1.05 (1.03, 1.07) | 1.05 (1.03, 1.07) | 1.05 (1.03, 1.07) | 1.05 (1.03, 1.08) |
| **International norms score** | 1.01 (0.97, 1.05) | 1.01 (0.96, 1.05) | 1.01 (0.97, 1.05) | 1.00 (0.96, 1.05) |
| **Risk environment score** | 1.15 (1.08, 1.23) | 1.15 (1.08, 1.23) | 1.15 (1.08, 1.23) | 1.16 (1.08, 1.23) |
| ***Joint External Evaluation (JEE) ready score*** | 1.16 (1.10, 1.22) | 1.16 (1.10, 1.22) | 1.16 (1.11, 1.22) | 1.16 (1.10, 1.22) |
| **Prevention score** | 1.16 (1.10, 1.22) | 1.16 (1.10, 1.22) | 1.16 (1.10, 1.22) | 1.15 (1.10, 1.21) |
| **Detection score** | 1.18 (1.10, 1.28) | 1.18 (1.10, 1.27) | 1.19 (1.11, 1.28) | 1.19 (1.10, 1.28) |
| **Response score** | 1.08 (1.03, 1.13) | 1.08 (1.03, 1.13) | 1.08 (1.04, 1.13) | 1.08 (1.04, 1.13) |
| NOTE: Effect sizes are rate ratios comparing 10% differences in each index. Individual regressions were implemented for each GHS Index measure to assess the effect of the measure independent of other indicators. Covariates included in each regression was log transformed gross domestic product (GDP) per capita. | | | | |

**Table S5**. Country-level effect sizes of pandemic preparedness capacities on COVID-19 testing rates stratified by time periods after first reported case in each country.

| **Pandemic preparedness capacity** | **Testing rate ratio** | | | | |
| --- | --- | --- | --- | --- | --- |
|  | **Day 100** | **Day 100 to Day 300** | **Day 300 to Day 500** | **Day 500 to Day 700** | **Complete observation period** |
| ***Global Health Security Index score*** | 1.00 (0.92, 1.08) | 0.98 (0.90, 1.06) | 0.98 (0.91, 1.07) | 1.01 (0.94, 1.08) | 1.03 (0.97, 1.10) |
| **Prevention score** | 1.03 (0.99, 1.07) | 1.04 (1.00, 1.09) | 1.05 (1.00, 1.09) | 1.02 (1.00, 1.05) | 1.03 (1.01, 1.05) |
| **Detection score** | 0.99 (0.96, 1.03) | 0.98 (0.95, 1.01) | 0.99 (0.96, 1.03) | 1.00 (0.98, 1.02) | 1.00 (0.98, 1.03) |
| **Response score** | 0.93 (0.87, 0.98) | 0.93 (0.87, 1.00) | 0.94 (0.88, 1.00) | 0.96 (0.89, 1.03) | 0.96 (0.90, 1.02) |
| **Health system score** | 1.03 (0.98, 1.08) | 1.01 (0.97, 1.06) | 1.00 (0.96, 1.04) | 1.02 (0.98, 1.06) | 1.02 (0.99, 1.06) |
| **International norms score** | 0.96 (0.90, 1.02) | 0.93 (0.88, 0.99) | 0.94 (0.89, 0.99) | 0.95 (0.90, 1.00) | 0.94 (0.90, 0.99) |
| **Risk environment score** | 1.09 (0.96, 1.23) | 1.16 (1.00, 1.34) | 1.11 (0.95, 1.28) | 1.12 (0.97, 1.31) | 1.26 (1.14, 1.40) |
| ***Joint External Evaluation (JEE) ready score*** | 1.07 (0.92, 1.25) | 1.04 (0.89, 1.21) | 1.12 (0.95, 1.32) | 1.09 (0.96, 1.22) | 1.11 (0.97, 1.26) |
| **Prevention score** | 1.06 (0.92, 1.22) | 1.05 (0.92, 1.20) | 1.13 (0.99, 1.31) | 1.07 (0.97, 1.18) | 1.09 (0.97, 1.23) |
| **Detection score** | 0.99 (0.82, 1.20) | 1.00 (0.79, 1.27) | 1.12 (0.89, 1.41) | 1.03 (0.88, 1.20) | 1.07 (0.93, 1.22) |
| **Response score** | 1.09 (0.99, 1.19) | 1.04 (0.94, 1.15) | 1.07 (0.95, 1.20) | 1.09 (0.99, 1.18) | 1.08 (1.00, 1.17) |
| NOTE: Effect sizes are rate ratios comparing 10% differences in each index. Individual regressions were implemented for each GHS Index measure to assess the effect of the measure independent of other indicators. Covariates included in each regression was log transformed gross domestic product (GDP) per capita. | | | | | |

**Table S6**. Country-level effect sizes of pandemic preparedness capacities on SARS-CoV-2 infection completion rates stratified by time periods after first reported global case.

| **Pandemic preparedness capacity** | **SARS-CoV-2 infection completion rate ratios** | | | | |
| --- | --- | --- | --- | --- | --- |
|  | **09 April 2020 Day 100** | **26 October 2020 Day 300** | **14 May 2021 Day 500** | **30 November 2021 Day 700** | **Complete observation period** |
| ***Global Health Security Index score*** | 1.21 (1.12, 1.30) | 1.12 (1.06, 1.18) | 1.13 (1.06, 1.19) | 1.14 (1.07, 1.23) | 1.13 (1.07, 1.20) |
| **Prevention score** | 1.10 (1.07, 1.12) | 1.06 (1.04, 1.08) | 1.07 (1.05, 1.10) | 1.08 (1.06, 1.11) | 1.08 (1.05, 1.10) |
| **Detection score** | 1.05 (1.02, 1.08) | 1.04 (1.01, 1.06) | 1.03 (1.00, 1.05) | 1.04 (1.01, 1.07) | 1.03 (1.01, 1.06) |
| **Response score** | 1.07 (0.98, 1.15) | 1.04 (0.98, 1.10) | 1.04 (0.97, 1.11) | 1.04 (0.96, 1.11) | 1.05 (0.98, 1.11) |
| **Health system score** | 1.10 (1.06, 1.14) | 1.06 (1.03, 1.09) | 1.07 (1.04, 1.10) | 1.06 (1.03, 1.10) | 1.06 (1.03, 1.09) |
| **International norms score** | 1.06 (0.99, 1.14) | 1.02 (0.96, 1.09) | 1.00 (0.95, 1.06) | 0.99 (0.93, 1.05) | 1.01 (0.95, 1.06) |
| **Risk environment score** | 1.29 (1.12, 1.48) | 1.18 (1.06, 1.31) | 1.22 (1.10, 1.36) | 1.32 (1.18, 1.49) | 1.27 (1.15, 1.41) |
| ***Joint External Evaluation (JEE) ready score*** | 1.42 (1.28, 1.57) | 1.17 (1.08, 1.27) | 1.13 (1.04, 1.22) | 1.19 (1.09, 1.31) | 1.18 (1.09, 1.28) |
| **Prevention score** | 1.38 (1.25, 1.52) | 1.17 (1.09, 1.25) | 1.15 (1.06, 1.25) | 1.21 (1.12, 1.32) | 1.20 (1.11, 1.30) |
| **Detection score** | 1.49 (1.26, 1.76) | 1.17 (1.04, 1.31) | 1.10 (0.97, 1.24) | 1.20 (1.05, 1.37) | 1.18 (1.05, 1.33) |
| **Response score** | 1.22 (1.11, 1.35) | 1.10 (1.03, 1.17) | 1.05 (0.98, 1.13) | 1.09 (1.00, 1.18) | 1.07 (1.00, 1.16) |
| NOTE: Effect sizes are rate ratios comparing 10% differences in each index. Individual regressions were implemented for each GHS Index measure to assess the effect of the measure independent of other indicators. Covariates included in each regression was log transformed gross domestic product (GDP) per capita. April 09 2020 was day 100 after first reported global case. Stratified regressions are specific to each time period (e.g., October 2020 regression [300 days] only included the completion percentage between April 2020 and October 2020 [100 to 300 days]). | | | | | |

**Table S7**. Country-level effect sizes of pandemic preparedness capacities on cumulative COVID-19 infection detection rate stratified by time period.

| **Pandemic preparedness capacity** | **SARS-CoV-2 infection completion rate ratio** | | | | |
| --- | --- | --- | --- | --- | --- |
|  | **30 June 2020** | **31 December 2020** | **30 June 2021** | **31 December 2021** | **Total observation period** |
| ***Global Health Security Index score*** | 1.13 (1.06, 1.19) | 1.14 (1.08, 1.20) | 1.11 (1.05, 1.18) | 1.17 (1.08, 1.26) | 1.14 (1.07, 1.22) |
| **Prevention score** | 1.07 (1.05, 1.09) | 1.07 (1.04, 1.09) | 1.07 (1.05, 1.10) | 1.09 (1.07, 1.12) | 1.08 (1.06, 1.11) |
| **Detection score** | 1.04 (1.02, 1.06) | 1.04 (1.01, 1.06) | 1.02 (1.00, 1.05) | 1.05 (1.01, 1.08) | 1.04 (1.01, 1.07) |
| **Response score** | 1.03 (0.97, 1.09) | 1.04 (0.98, 1.10) | 1.03 (0.97, 1.10) | 1.05 (0.97, 1.14) | 1.05 (0.98, 1.12) |
| **Health system score** | 1.07 (1.04, 1.10) | 1.07 (1.04, 1.10) | 1.06 (1.03, 1.09) | 1.06 (1.02, 1.11) | 1.06 (1.03, 1.09) |
| **International norms score** | 1.01 (0.95, 1.07) | 1.03 (0.97, 1.09) | 0.98 (0.93, 1.04) | 1.02 (0.96, 1.09) | 1.01 (0.95, 1.07) |
| **Risk environment score** | 1.15 (1.04, 1.26) | 1.20 (1.08, 1.34) | 1.23 (1.11, 1.36) | 1.34 (1.17, 1.53) | 1.27 (1.14, 1.42) |
| ***Joint External Evaluation (JEE) ready score*** | 1.23 (1.14, 1.34) | 1.17 (1.07, 1.27) | 1.11 (1.02, 1.21) | 1.26 (1.13, 1.40) | 1.19 (1.10, 1.30) |
| **Prevention score** | 1.21 (1.12, 1.30) | 1.17 (1.07, 1.27) | 1.13 (1.04, 1.23) | 1.29 (1.16, 1.43) | 1.22 (1.12, 1.33) |
| **Detection score** | 1.24 (1.09, 1.41) | 1.16 (1.03, 1.31) | 1.08 (0.95, 1.23) | 1.28 (1.10, 1.49) | 1.20 (1.05, 1.36) |
| **Response score** | 1.14 (1.07, 1.22) | 1.09 (1.02, 1.16) | 1.04 (0.97, 1.12) | 1.10 (1.01, 1.21) | 1.08 (1.00, 1.17) |
| NOTE: Effect sizes are rate ratios comparing 10% differences in each index. Individual regressions were implemented for each GHS Index measure to assess the effect of the measure independent of other indicators. Covariates included in each regression was log transformed gross domestic product (GDP) per capita. Stratified regressions are specific to each time period (e.g., December 2020 regression only included the completion percentage between June 2020 and December 2020). | | | | | |

**Table S8**. Country-level effect sizes of 2019 pandemic preparedness capacities on cumulative SARS-CoV-2 infection and death completion rates.

| **Pandemic preparedness capacity** | **SARS-CoV-2 infection completion rate ratio** | | **COVID-19 death completion rate ratio** | |
| --- | --- | --- | --- | --- |
|  | **Day 100** | **Day 700** | **Day 100** | **Day 700** |
| ***Global Health Security Index score*** | 1.08 (1.03, 1.15) | 1.13 (1.07, 1.20) | 1.09 (1.04, 1.14) | 1.09 (1.05, 1.14) |
| **Prevention score** | 1.05 (1.03, 1.07) | 1.08 (1.06, 1.11) | 1.05 (1.03, 1.07) | 1.05 (1.04, 1.07) |
| **Detection score** | 1.02 (1.00, 1.04) | 1.03 (1.00, 1.06) | 1.02 (1.01, 1.04) | 1.02 (1.00, 1.04) |
| **Response score** | 1.05 (0.99, 1.11) | 1.08 (1.02, 1.14) | 1.05 (1.01, 1.10) | 1.06 (1.01, 1.11) |
| **Health system score** | 1.05 (1.03, 1.08) | 1.06 (1.03, 1.09) | 1.04 (1.02, 1.07) | 1.05 (1.03, 1.07) |
| **International norms score** | 0.99 (0.94, 1.05) | 1.02 (0.96, 1.08) | 1.01 (0.96, 1.06) | 1.01 (0.96, 1.06) |
| **Risk environment score** | 1.21 (1.10, 1.34) | 1.29 (1.16, 1.45) | 1.16 (1.07, 1.25) | 1.15 (1.07, 1.24) |
| ***Joint External Evaluation (JEE) ready score*** | 1.17 (1.07, 1.27) | 1.19 (1.10, 1.30) | 1.16 (1.10, 1.22) | 1.16 (1.10, 1.22) |
| **Prevention score** | 1.17 (1.08, 1.26) | 1.21 (1.12, 1.32) | 1.16 (1.10, 1.22) | 1.15 (1.10, 1.21) |
| **Detection score** | 1.14 (1.00, 1.29) | 1.23 (1.09, 1.39) | 1.18 (1.10, 1.28) | 1.19 (1.10, 1.28) |
| **Response score** | 1.10 (1.02, 1.18) | 1.08 (1.01, 1.16) | 1.08 (1.03, 1.13) | 1.08 (1.04, 1.13) |

**Table S9**. Country-level effect sizes of pandemic preparedness capacities on COVID-19 burden adjusted for COVID-19 response stringency.

| **Pandemic preparedness capacity** | **SARS-CoV-2 infection rate ratios** | | **Age-standardized COVID-19 death rate ratios** | |
| --- | --- | --- | --- | --- |
|  | **Day 100** | **Day 300** | **Day 100** | **Day 300** |
| ***Global Health Security Index score*** | 0.86 (0.78, 0.95) | 0.90 (0.82, 0.99) | 0.85 (0.75, 0.96) | 0.89 (0.79, 1.00) |
| **Prevention score** | 0.94 (0.90, 0.98) | 0.97 (0.93, 1.00) | 0.93 (0.89, 0.98) | 0.97 (0.93, 1.01) |
| **Detection score** | 0.95 (0.91, 0.99) | 0.96 (0.92, 1.00) | 0.94 (0.90, 0.99) | 0.95 (0.90, 1.01) |
| **Response score** | 0.92 (0.84, 1.02) | 0.93 (0.86, 1.01) | 0.92 (0.81, 1.04) | 0.91 (0.81, 1.03) |
| **Health system score** | 0.97 (0.93, 1.01) | 0.99 (0.96, 1.03) | 0.96 (0.91, 1.01) | 0.99 (0.94, 1.04) |
| **International norms score** | 0.91 (0.84, 0.99) | 0.93 (0.87, 1.00) | 0.94 (0.85, 1.04) | 0.94 (0.87, 1.03) |
| **Risk environment score** | 0.82 (0.72, 0.92) | 0.87 (0.80, 0.93) | 0.72 (0.62, 0.84) | 0.84 (0.76, 0.93) |

NOTE: Effect sizes are rate ratios comparing 10% differences in each index. Individual regressions were implemented for each GHS Index measure to assess the effect of the measure independent of other indicators. Covariates included in each regression was log transformed gross domestic product (GDP) per capita and the stringency index.

**Table S10**. Country-level effect sizes of pandemic preparedness capacities on cumulative SARS-CoV-2 infection and death completion rates stratified by World Bank income groups at 700 days after first reported case in each country.

| **Pandemic preparedness capacity** | **Low Income** | **Lower Middle Income** | **Upper Middle Income** | **High Income** |
| --- | --- | --- | --- | --- |
| **SARS-CoV-2 infection completion rate ratio at 700 days** | | | | |
| **Global Health Security Index score** | 1.18 (1.04, 1.34) | 1.41 (1.16, 1.71) | 1.13 (1.04, 1.23) | 1.13 (1.05, 1.21) |
| Prevention score | 1.00 (0.86, 1.16) | 1.12 (1.04, 1.20) | 1.06 (1.04, 1.08) | 1.09 (1.04, 1.16) |
| Detection score | 1.04 (0.94, 1.15) | 1.09 (1.02, 1.17) | 1.05 (1.02, 1.08) | 1.02 (0.99, 1.05) |
| Response score | 1.08 (0.90, 1.31) | 1.11 (0.94, 1.32) | 1.13 (1.02, 1.25) | 1.10 (1.02, 1.18) |
| Health system score | 1.05 (1.00, 1.09) | 1.13 (1.06, 1.20) | 1.05 (1.00, 1.10) | 1.05 (1.00, 1.10) |
| International norms score | 1.00 (0.87, 1.14) | 0.88 (0.76, 1.02) | 1.12 (0.99, 1.26) | 1.05 (1.00, 1.10) |
| Risk environment score | 1.34 (1.18, 1.52) | 1.45 (1.14, 1.84) | 1.25 (1.10, 1.42) | 1.35 (1.14, 1.60) |
| **Joint External Evaluation (JEE) ready score** | 1.24 (1.06, 1.44) | 1.44 (1.21, 1.71) | 1.17 (1.05, 1.32) | 1.37 (0.96, 1.95) |
| Prevention score | 1.26 (1.11, 1.44) | 1.50 (1.26, 1.78) | 1.16 (1.06, 1.26) | 1.43 (1.06, 1.93) |
| Detection score | 1.22 (0.95, 1.55) | 1.50 (1.19, 1.90) | 1.30 (1.12, 1.50) | 1.38 (0.99, 1.94) |
| Response score | 1.08 (0.97, 1.21) | 1.24 (1.11, 1.39) | 1.11 (1.00, 1.24) | 0.95 (0.72, 1.24) |
| **COVID-19 death completion rate ratio at 700 days** | | | | |
| **Global Health Security Index score** | 1.19 (1.09, 1.31) | 1.25 (1.10, 1.43) | 1.14 (1.08, 1.20) | 1.02 (0.97, 1.07) |
| Prevention score | 1.03 (0.95, 1.10) | 1.07 (1.02, 1.12) | 1.06 (1.04, 1.07) | 1.02 (0.99, 1.05) |
| Detection score | 1.03 (0.96, 1.11) | 1.07 (1.02, 1.12) | 1.04 (1.02, 1.06) | 1.00 (0.99, 1.02) |
| Response score | 1.17 (1.05, 1.29) | 1.04 (0.92, 1.18) | 1.13 (1.06, 1.21) | 1.02 (0.97, 1.07) |
| Health system score | 1.04 (1.00, 1.07) | 1.09 (1.03, 1.15) | 1.06 (1.02, 1.10) | 1.02 (0.99, 1.05) |
| International norms score | 1.08 (1.01, 1.15) | 0.93 (0.83, 1.05) | 1.10 (1.00, 1.20) | 1.00 (0.96, 1.03) |
| Risk environment score | 1.23 (1.16, 1.30) | 1.29 (1.07, 1.56) | 1.20 (1.10, 1.32) | 1.05 (0.96, 1.14) |
| **Joint External Evaluation (JEE) ready score** | 1.18 (1.10, 1.27) | 1.29 (1.14, 1.45) | 1.17 (1.09, 1.26) | 1.14 (0.93, 1.41) |
| Prevention score | 1.16 (1.09, 1.24) | 1.32 (1.17, 1.49) | 1.15 (1.09, 1.22) | 1.13 (0.93, 1.37) |
| Detection score | 1.15 (1.00, 1.32) | 1.37 (1.16, 1.61) | 1.28 (1.16, 1.42) | 1.19 (1.03, 1.37) |
| Response score | 1.10 (1.05, 1.16) | 1.16 (1.08, 1.26) | 1.11 (1.03, 1.20) | 0.94 (0.72, 1.23) |

**Table S11**. Country-level effect sizes of pandemic preparedness capacities on cumulative SARS-CoV-2 infection rates stratified by World Bank income groups during the pre-vaccine era.

| **Pandemic preparedness capacity** | **Low Income** | **Lower Middle Income** | **Upper Middle Income** | **High Income** |
| --- | --- | --- | --- | --- |
| **SARS-CoV-2 infection rate ratio at 100 days** | | | | |
| **Global Health Security Index score** | 0.79 (0.70, 0.88) | 0.73 (0.58, 0.91) | 0.91 (0.77, 1.08) | 0.89 (0.76, 1.04) |
| Prevention score | 0.91 (0.81, 1.02) | 0.93 (0.87, 1.00) | 0.95 (0.91, 1.00) | 0.92 (0.82, 1.03) |
| Detection score | 0.90 (0.82, 0.99) | 0.89 (0.82, 0.97) | 0.96 (0.91, 1.02) | 0.97 (0.92, 1.03) |
| Response score | 0.86 (0.74, 1.00) | 0.89 (0.75, 1.07) | 0.90 (0.75, 1.07) | 0.94 (0.83, 1.08) |
| Health system score | 0.95 (0.91, 0.98) | 0.97 (0.90, 1.03) | 0.98 (0.90, 1.07) | 0.97 (0.89, 1.06) |
| International norms score | 0.92 (0.84, 1.02) | 0.93 (0.78, 1.10) | 0.97 (0.79, 1.18) | 0.86 (0.77, 0.96) |
| Risk environment score | 0.81 (0.69, 0.95) | 0.85 (0.63, 1.15) | 0.73 (0.58, 0.93) | 0.95 (0.75, 1.19) |
| **Joint External Evaluation (JEE) ready score** | 0.77 (0.67, 0.89) | 0.73 (0.59, 0.90) | 0.79 (0.59, 1.06) | 0.41 (0.22, 0.74) |
| Prevention score | 0.78 (0.70, 0.87) | 0.67 (0.54, 0.83) | 0.82 (0.65, 1.03) | 0.42 (0.28, 0.63) |
| Detection score | 0.73 (0.65, 0.83) | 0.67 (0.52, 0.88) | 0.69 (0.49, 0.99) | 0.52 (0.30, 0.90) |
| Response score | 0.90 (0.81, 1.00) | 0.83 (0.72, 0.96) | 0.85 (0.67, 1.07) | 1.02 (0.55, 1.88) |
| **SARS-CoV-2 infection rate ratio at 300 days** | | | | |
| **Global Health Security Index score** | 0.89 (0.83, 0.94) | 0.81 (0.64, 1.01) | 0.94 (0.81, 1.11) | 0.86 (0.75, 0.98) |
| Prevention score | 0.97 (0.92, 1.01) | 0.95 (0.90, 1.02) | 0.98 (0.94, 1.02) | 0.92 (0.85, 1.00) |
| Detection score | 0.93 (0.90, 0.97) | 0.92 (0.85, 0.99) | 0.99 (0.93, 1.05) | 0.95 (0.90, 1.00) |
| Response score | 0.94 (0.88, 0.99) | 0.97 (0.87, 1.08) | 0.89 (0.76, 1.05) | 0.88 (0.79, 0.98) |
| Health system score | 0.98 (0.96, 0.99) | 0.99 (0.95, 1.03) | 1.00 (0.92, 1.08) | 0.97 (0.91, 1.05) |
| International norms score | 0.96 (0.91, 1.01) | 0.93 (0.83, 1.03) | 1.03 (0.83, 1.27) | 0.88 (0.80, 0.96) |
| Risk environment score | 0.89 (0.83, 0.96) | 0.88 (0.70, 1.10) | 0.73 (0.60, 0.89) | 0.83 (0.70, 0.98) |
| **Joint External Evaluation (JEE) ready score** | 0.88 (0.83, 0.94) | 0.84 (0.70, 1.02) | 0.82 (0.62, 1.09) | 0.33 (0.22, 0.51) |
| Prevention score | 0.90 (0.85, 0.95) | 0.78 (0.61, 1.00) | 0.85 (0.67, 1.07) | 0.43 (0.31, 0.60) |
| Detection score | 0.86 (0.80, 0.93) | 0.81 (0.64, 1.02) | 0.76 (0.53, 1.09) | 0.40 (0.26, 0.60) |
| Response score | 0.94 (0.89, 0.98) | 0.93 (0.84, 1.03) | 0.88 (0.71, 1.07) | 0.63 (0.37, 1.06) |
| NOTE: Effect sizes are rate ratios comparing 10% differences in each index. Individual regressions were implemented for each GHS Index measure to assess the effect of the measure independent of other indicators. No adjustment for GDP per capita in regressions as stratification is a form of covariate adjustment. | | | | |

**Table S12**. Country-level effect sizes of pandemic preparedness capacities on cumulative age-standardized COVID-19 death rates stratified by World Bank income groups during the pre-vaccine era.

| **Pandemic preparedness capacity** | **Low Income** | **Lower Middle Income** | **Upper Middle Income** | **High Income** |
| --- | --- | --- | --- | --- |
| **Age-standardized COVID-19 death rate ratio at 100 days** | | | | |
| **Global Health Security Index score** | 0.75 (0.62, 0.90) | 0.65 (0.49, 0.85) | 0.89 (0.72, 1.09) | 0.92 (0.76, 1.11) |
| Prevention score | 0.93 (0.81, 1.08) | 0.92 (0.85, 0.98) | 0.93 (0.88, 0.99) | 0.97 (0.85, 1.10) |
| Detection score | 0.88 (0.76, 1.02) | 0.85 (0.77, 0.94) | 0.95 (0.88, 1.02) | 0.97 (0.90, 1.05) |
| Response score | 0.81 (0.62, 1.06) | 0.89 (0.74, 1.06) | 0.92 (0.72, 1.18) | 0.93 (0.80, 1.09) |
| Health system score | 0.94 (0.89, 1.00) | 0.94 (0.86, 1.02) | 0.97 (0.87, 1.08) | 0.95 (0.86, 1.06) |
| International norms score | 0.96 (0.82, 1.13) | 0.91 (0.73, 1.15) | 0.94 (0.72, 1.21) | 0.90 (0.80, 1.02) |
| Risk environment score | 0.65 (0.55, 0.77) | 0.72 (0.49, 1.06) | 0.66 (0.50, 0.87) | 1.09 (0.82, 1.45) |
| **Joint External Evaluation (JEE) ready score** | 0.82 (0.65, 1.04) | 0.70 (0.51, 0.95) | 0.81 (0.58, 1.13) | 0.49 (0.24, 1.00) |
| Prevention score | 0.78 (0.66, 0.93) | 0.65 (0.49, 0.85) | 0.84 (0.65, 1.09) | 0.41 (0.27, 0.62) |
| Detection score | 0.78 (0.55, 1.11) | 0.56 (0.39, 0.82) | 0.67 (0.45, 0.99) | 0.52 (0.27, 0.99) |
| Response score | 0.96 (0.83, 1.11) | 0.83 (0.67, 1.02) | 0.86 (0.66, 1.13) | 1.66 (0.91, 3.00) |
| **Age-standardized COVID-19 death rate ratio at 300 days** | | | | |
| **Global Health Security Index score** | 0.90 (0.80, 1.01) | 0.78 (0.60, 1.01) | 0.91 (0.74, 1.12) | 0.87 (0.75, 1.01) |
| Prevention score | 0.99 (0.94, 1.05) | 0.97 (0.90, 1.04) | 0.96 (0.92, 1.01) | 0.95 (0.87, 1.03) |
| Detection score | 0.91 (0.86, 0.96) | 0.90 (0.82, 0.98) | 0.98 (0.91, 1.06) | 0.96 (0.91, 1.01) |
| Response score | 0.91 (0.81, 1.03) | 0.99 (0.87, 1.12) | 0.85 (0.68, 1.08) | 0.85 (0.74, 0.97) |
| Health system score | 0.99 (0.96, 1.02) | 0.98 (0.92, 1.04) | 0.98 (0.89, 1.09) | 0.98 (0.89, 1.06) |
| International norms score | 0.97 (0.90, 1.06) | 0.89 (0.78, 1.02) | 1.02 (0.76, 1.37) | 0.91 (0.82, 1.00) |
| Risk environment score | 0.91 (0.83, 1.00) | 0.85 (0.63, 1.14) | 0.66 (0.53, 0.82) | 0.76 (0.61, 0.96) |
| **Joint External Evaluation (JEE) ready score** | 0.93 (0.85, 1.02) | 0.85 (0.66, 1.08) | 0.77 (0.53, 1.13) | 0.31 (0.16, 0.61) |
| Prevention score | 0.94 (0.86, 1.02) | 0.79 (0.58, 1.08) | 0.80 (0.57, 1.11) | 0.37 (0.21, 0.65) |
| Detection score | 0.84 (0.78, 0.91) | 0.75 (0.55, 1.02) | 0.66 (0.41, 1.07) | 0.33 (0.19, 0.57) |
| Response score | 0.99 (0.93, 1.05) | 0.95 (0.84, 1.07) | 0.85 (0.65, 1.10) | 0.82 (0.41, 1.64) |
| NOTE: Effect sizes are rate ratios comparing 10% differences in each index. Individual regressions were implemented for each GHS Index measure to assess the effect of the measure independent of other indicators. No adjustment for GDP per capita in regressions as stratification is a form of covariate adjustment. | | | | |

**Table S13.** Country-level effect sizes of pandemic preparedness capacities on cumulative SARS-CoV-2 infection rates and age-standardized COVID-19 deaths at 100 and 300 days [using non-robust standard errors]

| **Pandemic preparedness capacity** | **SARS-CoV-2 infection rate ratios** | | **Age-standardized COVID-19 death rate ratios** | |
| --- | --- | --- | --- | --- |
|  | **Day 100** | **Day 300** | **Day 100** | **Day 300** |
| ***Global Health Security Index score*** | 0.86 (0.79, 0.94) | 0.91 (0.84, 0.98) | 0.84 (0.75, 0.95) | 0.90 (0.82, 0.98) |
| **Prevention score** | 0.94 (0.91, 0.98) | 0.97 (0.94, 1.00) | 0.93 (0.89, 0.98) | 0.97 (0.93, 1.01) |
| **Detection score** | 0.95 (0.92, 0.98) | 0.96 (0.94, 0.99) | 0.94 (0.90, 0.98) | 0.96 (0.93, 0.99) |
| **Response score** | 0.92 (0.85, 1.01) | 0.93 (0.86, 1.00) | 0.92 (0.82, 1.03) | 0.91 (0.83, 0.99) |
| **Health system score** | 0.97 (0.93, 1.01) | 0.99 (0.96, 1.03) | 0.95 (0.90, 1.01) | 0.99 (0.95, 1.04) |
| **International norms score** | 0.91 (0.84, 0.98) | 0.93 (0.87, 0.99) | 0.93 (0.85, 1.03) | 0.94 (0.87, 1.02) |
| **Risk environment score** | 0.82 (0.71, 0.92) | 0.86 (0.77, 0.97) | 0.72 (0.61, 0.85) | 0.84 (0.72, 0.97) |

**References**

1. Ravi SJ, Warmbrod KL, Mullen L, Meyer D, Cameron E, Bell J, et al. The value proposition of the Global Health Security Index. BMJ Glob Health. 2020 Oct 8;5(10):e003648.

2. COVID-19 Forecasting Team. Variation in the COVID-19 infection-fatality ratio by age, time, and geography during the pre-vaccine era: a systematic analysis. The Lancet . 2022;399(10334):1469–88.

3. Hradsky O, Komarek A. Demographic and public health characteristics explain large part of variability in COVID-19 mortality across countries. Eur J Public Health. 2021 Feb 1;31(1):12–6.

4. Dowd JB, Andriano L, Brazel DM, Rotondi V, Block P, Ding X, et al. Demographic science aids in understanding the spread and fatality rates of COVID-19. Proceedings of the National Academy of Sciences. 2020 May 5;117(18):9696–8.

5. Bauer P, Brugger J, König F, Posch M. An international comparison of age and sex dependency of COVID-19 deaths in 2020: a descriptive analysis. Sci Rep. 2021 Dec 27;11(1):19143.

6. Heuveline P, Tzen M. Beyond deaths per capita: comparative COVID-19 mortality indicators. BMJ Open. 2021 Mar 10;11(3):e042934.

7. Caporali A, Garcia J, Couppié É, Poniakina S, Barbieri M, Bonnet F, et al. The demography of COVID-19 deaths database, a gateway to well-documented international data. Sci Data. 2022 Dec 22;9(1):93.
